# Deep immune profiling reveals targetable mechanisms of immune evasion in immune checkpoint inhibitor-refractory glioblastoma

**DOI:** 10.1136/jitc-2020-002181

**Published:** 2021-06-02

**Authors:** Erin F Simonds, Edbert D Lu, Oscar Badillo, Shokoufeh Karimi, Eric V Liu, Whitney Tamaki, Chiara Rancan, Kira M Downey, Jacob Stultz, Meenal Sinha, Lauren K McHenry, Nicole M Nasholm, Pavlina Chuntova, Anders Sundström, Vassilis Genoud, Shilpa A Shahani, Leo D Wang, Christine E Brown, Paul R Walker, Fredrik J Swartling, Lawrence Fong, Hideho Okada, William A Weiss, Mats Hellström

**Affiliations:** 1 Department of Neurology, University of California San Francisco, San Francisco, California, USA; 2 Department of Immunology, Genetics and Pathology, Uppsala University, Uppsala, Sweden; 3 Division of Hematology/Oncology, Department of Medicine, University of California San Francisco, San Francisco, California, USA; 4 Department of Neurological Surgery, University of California San Francisco, San Francisco, California, USA; 5 Translational Research Centre in Oncohaematology, Department of Medicine, University of Geneva, Geneva, Switzerland; 6 Department of Pediatrics, City of Hope National Medical Center, Duarte, California, USA; 7 Department of Immuno-Oncology, City of Hope National Medical Center, Duarte, California, USA; 8 Departments of Hematology and Hematopoietic Cell Transplantation, City of Hope National Medical Center, Duarte CA, Duarte, California, USA; 9 Parker Institute for Cancer Immunotherapy, San Francisco, California, USA; 10 Departments of Neurology, Neurological Surgery, and Pediatrics, University of California San Francisco, San Francisco, California, USA; 11 Helen Diller Family Comprehensive Cancer Center, University of California San Francisco, San Francisco, California, USA

**Keywords:** biomarkers, tumor, brain neoplasms, dendritic cells, immunotherapy, tumor microenvironment

## Abstract

**Background:**

Glioblastoma (GBM) is refractory to immune checkpoint inhibitor (ICI) therapy. We sought to determine to what extent this immune evasion is due to intrinsic properties of the tumor cells versus the specialized immune context of the brain, and if it can be reversed.

**Methods:**

We used CyTOF mass cytometry to compare the tumor immune microenvironments (TIME) of human tumors that are generally ICI-refractory (GBM and sarcoma) or ICI-responsive (renal cell carcinoma), as well as mouse models of GBM that are ICI-responsive (GL261) or ICI-refractory (SB28). We further compared SB28 tumors grown intracerebrally versus subcutaneously to determine how tumor site affects TIME and responsiveness to dual CTLA-4/PD-1 blockade. Informed by these data, we explored rational immunotherapeutic combinations.

**Results:**

ICI-sensitivity in human and mouse tumors was associated with increased T cells and dendritic cells (DCs), and fewer myeloid cells, in particular PD-L1+ tumor-associated macrophages. The SB28 mouse model of GBM responded to ICI when grown subcutaneously but not intracerebrally, providing a system to explore mechanisms underlying ICI resistance in GBM. The response to ICI in the subcutaneous SB28 model required CD4 T cells and NK cells, but not CD8 T cells. Recombinant FLT3L expanded DCs, improved antigen-specific T cell priming, and prolonged survival of mice with intracerebral SB28 tumors, but at the cost of increased Tregs. Targeting PD-L1 also prolonged survival, especially when combined with stereotactic radiation.

**Conclusions:**

Our data suggest that a major obstacle for effective immunotherapy of GBM is poor antigen presentation in the brain, rather than intrinsic immunosuppressive properties of GBM tumor cells. Deep immune profiling identified DCs and PD-L1+ tumor-associated macrophages as promising targetable cell populations, which was confirmed using therapeutic interventions in vivo.

## Background

Glioblastoma (GBM) is the most common primary malignant brain tumor. Despite progress in understanding the genetics and biology of GBM, the prognosis remains dismal and treatment options are limited beyond maximal safe resection, radio-chemotherapy and tumor treating fields, resulting in a median survival of less than 2 years from diagnosis.^1^

Stimulation of the adaptive immune response using immune checkpoint inhibitor (ICI) drugs has revolutionized treatment for melanoma, lung, bladder, and renal cancers.^2^ The clinically approved ICI drugs target two classes of lymphocyte inhibitory pathways (CTLA-4, PD-1/PD-L1), which are particularly important in regulating priming and activation of T cells. Several clinical trials have evaluated the efficacy of ICI in GBM, but outcomes have been generally negative,^3 4^ even in somatically hypermutated GBM.^5^

The lack of clinically meaningful responses to ICI in GBM has been attributed to the specialized immune environment of the brain, which includes mechanical obstruction at the blood-brain-barrier, unique brain-resident phagocytes (microglia and border-associated macrophages), unconventional lymphatic drainage, and a generally immunosuppressive environment.^6 7^ However, the clinical failures of dual CTLA-4/PD-1 blockade in GBM cannot be entirely attributed to the immune specialization of the brain, as brain metastases from melanoma and non-small-cell lung cancer are ICI-responsive.^8^ Why, then, do gliomas resist immune checkpoint blockade, while brain metastases from melanomas and carcinomas generally respond?

Recently, two groups performed single-cell profiling of the tumor immune microenvironment (TIME) of primary brain malignancies and brain metastases to explore whether tumor origin influences immune cell recruitment.^9 10^ They showed that the TIME was shaped by the origin of the primary tumor—gliomas and ependymomas had reduced lymphocyte infiltration and more tumor-associated macrophages (TAMs), compared with melanoma or carcinoma brain metastases. TAMs were also skewed phenotypically based on their ontogeny as tissue-resident microglia or blood-derived monocytic macrophages, as well as the origin of the primary tumor. Brain tumors, both primary and metastatic, had uniformly poor infiltration of dendritic cells (DCs). These immune profiling efforts suggest that the myeloid cells in brain tumors are shaped by origin of the primary tumor, and that they can support a productive immune response in the context of carcinoma metastases, but not in GBM.

Thus, evidence in humans suggests tumor-intrinsic and tumor-extrinsic factors prevent effective immunotherapy of GBM. Tumor-intrinsic properties associated with the tissue origin of glioma affect the quantity and quality of the TIME. However, the extent to which tumor-extrinsic properties of the brain—unique cell types and poor antigen drainage to lymph nodes—influence T cell priming has not been addressed in the context of response to immune checkpoint blockade. Here, we combine mass cytometry profiling of human and mouse tumors to reveal that the makeup of the TIME is influenced by the origin of the primary tumor, as well as the site of growth. Furthermore, we show that certain mechanisms mediating intracerebral immune evasion can be targeted to improve survival.

## Methods

See online supplemental methods.

10.1136/jitc-2020-002181.supp1 Supplementary data

## Results

### ICI-refractory GBM is associated with abundant PD-L1+ tumor-associated macrophages and poor T cell infiltration

**Figure 1:**
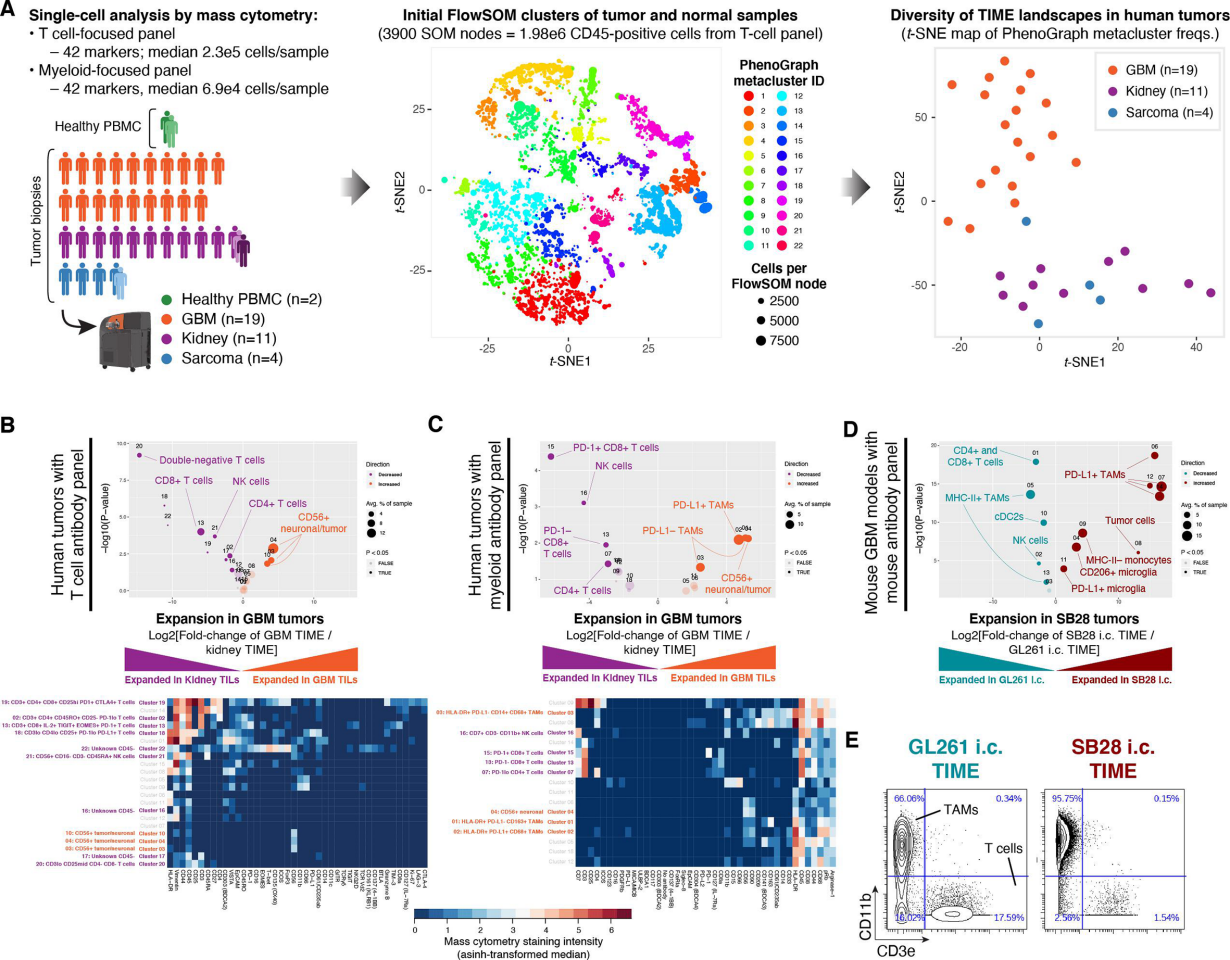
Abundant PD-L1+ tumor-associated macrophages and lack of MHC-II+ antigen-presenting cells are associated with resistance to dual CTLA-4/PD-1 checkpoint blockade. (A) Immune profiles were obtained from 39 samples: 19 glioblastoma (GBM) primary tumor biopsies, 11 renal cell carcinoma (RCC) primary tumor biopsies (one with paired tumor-adjacent normal tissue and metastatic lesion), 4 sarcoma primary tumor biopsies (one with paired tumor-adjacent normal tissue) and peripheral blood mononuclear cells (PBMC) from one healthy donor (with paired unstimulated and phytohemagglutinin-stimulated aliquots). Single-cell mass cytometry data were acquired using T cell-focused and myeloid-focused antibody panels with 42 markers each (see the Materials and methods section and online supplemental tables S2 and S3). Data were filtered on CD45-positive cells and processed using the PhenoGraph+FlowSOM analysis pipeline to segregate immune cell types into metaclusters and quantify their frequency across samples. To compare the overall tumor immune microenvironment (TIME) landscape across patients, metacluster frequencies for each primary tumor were fed into the *t*-stochastic neighbor embedding (*t*-SNE) dimensionality reduction algorithm to produce a map of TIME landscapes, organized spatially by similarity. Data from the T cell panel are shown in all plots; corresponding plots for the myeloid panel are in online supplemental figure S1A, B. (B) Volcano plot comparing abundance of immune cell populations (clusters) in GBM (orange) versus RCC (purple) tumor biopsies stained with the CyTOF human T-cell antibody panel. Statistically significant clusters in volcano plots are highlighted in opaque color and indicated with a cell type label. Diameter of the circle indicates the mean frequency of cells in the sample assigned to that cluster. Heatmap indicates manually annotatedcluster phenotypes and median intensity of antibody staining in each cluster. (C) Volcano plot and heatmap as in (B), stained with the CyTOF human myeloid antibody panel. (D) Volcano plot as in (B) comparing abundance of immune cell populations in mouse GBM models SB28 (red) and GL261 (teal) stained with the CyTOF mouse antibody panel. (E) Biaxial plots of representative raw CyTOF single-cell measurements of CD11b and CD3e on dissociated CD45+ cells from GL261 or SB28 tumors. These represent two of the 42 CyTOF mass cytometry channels, and two of the tumor biopsies, used to produce the volcano plot in (D). The staining patterns typical of tumor-associated macrophages (CD11b+), T-cells (CD3e+), and tumor cells (CD11b− CD3e−) are indicated, but clustering was performed using a total of 38 antibody markers (see online supplemental table S4).

We compared the immune cell populations in two ICI-refractory tumor types, GBM and sarcoma, to renal cell carcinoma (RCC), which is often ICI-responsive. Single-cell profiling of 19 GBM, 11 RCC, and 4 sarcoma tumors was performed using mass cytometry^11^ (figure 1A, online supplemental table S1). To improve the unsupervised detection of immune cell subsets, we included samples of healthy peripheral blood mononunclear cells (PBMC; PHA-stimulated and non-stimulated) from one normal donor, as well as paired normal tumor-adjacent tissue from one patient with RCC and one sarcoma patient. Samples were stained with two antibody panels focusing on either T cells or myeloid cells, each measuring 42 markers (online supplemental tables S2 and S3). Single-cell data were processed with the FlowSOM^12^ and PhenoGraph^13^ clustering algorithms to reduce the complexity from millions of CD45-positive cells to 22 metaclusters representing phenotypically distinct immune cell subsets (details in the Materials and methods section). Immune subset (eg, cluster) abundances from the T cell-focused staining panel were visualized as a reduced-dimensionality map using the *t*-SNE algorithm.^14 15^ In this high-level view of TIME landscapes across the three tumor types, all GBM samples were clearly separated from RCC and sarcoma, which were intermingled (figure 1A and online supplemental figure S1A, B). To explore this in more detail, we directly compared the immune cell subset abundances in GBM versus RCC using the edgeR algorithm.^16^ We found that nearly all subsets of T cells, plus NK cells, were decreased in the GBM TIME (figure 1B), consistent with prior reports.^17^ Using a second, myeloid-focused staining panel to compare GBM to RCC, we observed an increase in multiple TAM subsets in GBM, including a large population of PD-L1+ CD68+ TAMs (figure 1C). This is consistent with a report that a PD-L1+ myeloid cell subset was specific to GBM as compared with renal cell, colorectal, prostate, and non-small cell lung cancer.^17^

10.1136/jitc-2020-002181.supp2 Supplementary data

10.1136/jitc-2020-002181.supp3 Supplementary data

TIME profiling of primary tumors of different origins is confounded by their anatomical location. To directly compare ICI-responsive and ICI-refractory brain tumors, we turned to mouse models. The most commonly used mouse model of glioma, GL261, was established on the C57BL/6 background 50 years ago by chemical mutagenesis and was initially described as resembling ependymoblastoma by histology.^18^ The SB28 mouse model of invasive glioma was developed in 2014 through simultaneous suppression of p53, overexpression of *Pdgfb* and hyperactive ERK signaling through *NRAS^G12D^*, also on the C57BL/6 background.^19^ We analyzed bulk RNAseq datasets from in vivo orthotopic (intracerebral) SB28 and GL261 tumors and compared these to a reference cohort of human primary brain tumors. This cross-species analysis revealed that both SB28 and GL261 tumors were closely related to the mesenchymal subtype of GBM, and were distinct from other human primary brain tumors (online supplemental figure S1D, E). The two models differ markedly in response to ICI. GL261 responds to immunotherapies targeting a range of immune checkpoints including CTLA-4, PD-1, PD-L1, and TIM-3.^20 21^ SB28, on the other hand, does not respond to dual CTLA-4/PD-1 blockade, and has nearly 50-fold fewer non-synonymous mutations than GL261.^22^ We hypothesized that the increased mutation burden of GL261 would correspond to a more inflammatory phenotype in untreated tumors. Using Gene Set Enrichment Analysis,^23^ we observed a signature of increased interferon (IFN)-γ signaling in GL261 versus SB28 untreated tumors (online supplemental figure S1F). Increased IFN-γ signaling is associated with a more favorable environment for PD-1 blockade.^24^ Profiling the TIME of these two glioma models by mass cytometry (online supplemental table S4), we found that the ICI-resistant SB28 model had fewer T cells and cDC2s (cluster 10), as well as more PD-L1+ TAMs than the ICI-responsive GL261 model (figure 1D). Notably, the macrophage-rich, T cell-poor immune profile of SB28 was consistent with human GBM tumors (figure 1C). Among CD45+ cells in SB28 tumors, TAMs outnumbered T cells by 62:1, compared with 4:1 in GL261 tumors (figure D), indicating markedly different states of immune activation.

To compare TAM infiltration and PD-L1 expression in human GBM versus a wide range of other cancers, we analyzed the TCGA database for mRNA expression of two TAM-associated genes (*CD163*; PD-L1 *[CD274]*) and one T cell-associated gene (*CD3E*) (online supplemental figure S1G–I). GBM stood out as having the highest CD163/CD3E ratio of the 30 tumor types analyzed, indicating GBM tumors have a uniquely TAM-dominated TIME combined with poor T cell infiltration.

Overall, profiling the immune landscapes of human and mouse tumors highlighted the scarcity of T cells and plenitude of PD-L1+ TAMs in GBM. In humans, these features distinguished GBM from ICI-responsive RCC, while in mice, these same features distinguished ICI-refractory SB28 from ICI-responsive GL261.

### Response to dual CTLA-4/PD-1 blockade in SB28 is dependent on tumor site and correlated with abundance of DCs

**Figure 2:**
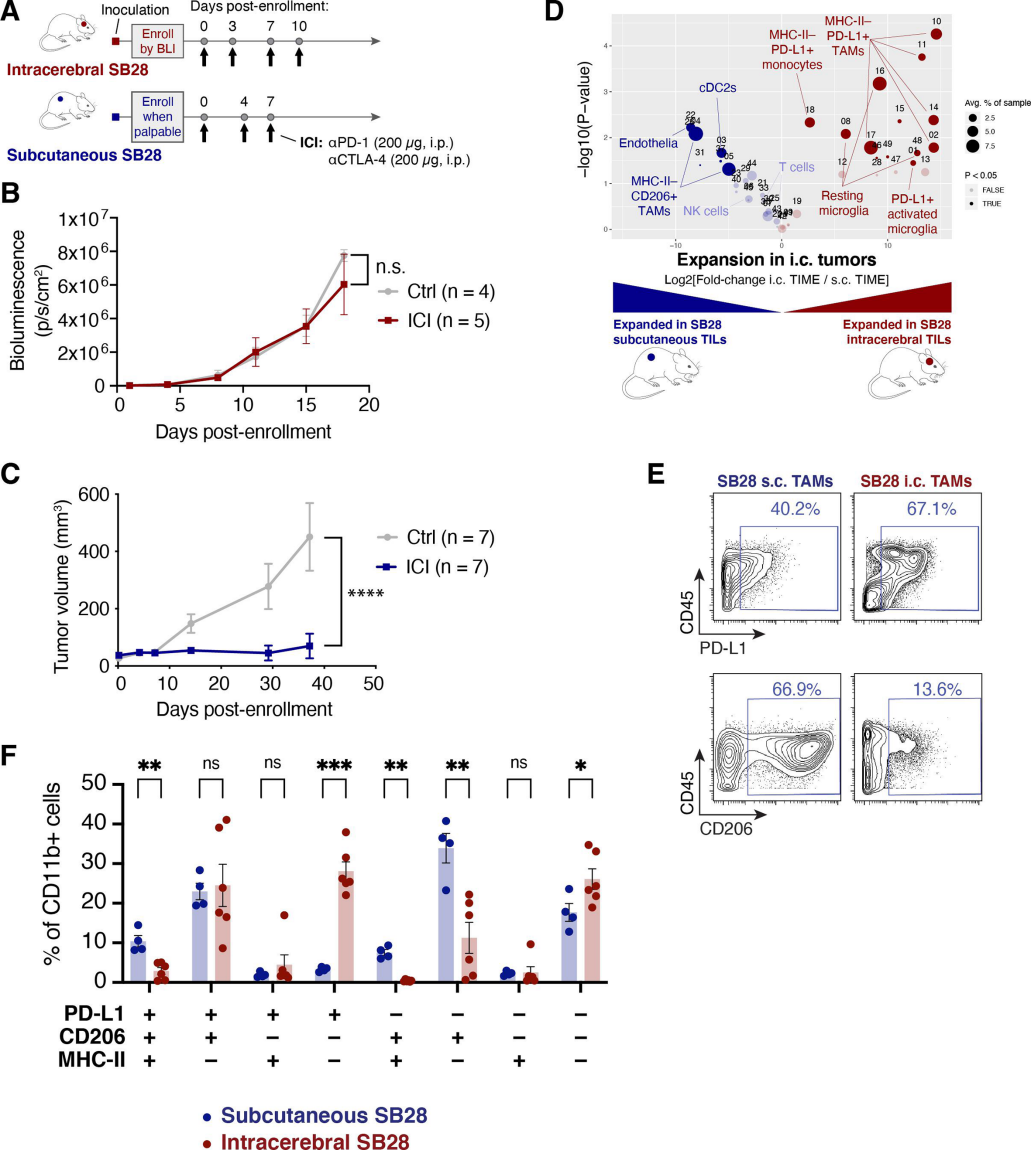
Subcutaneous SB28 tumors differ from intracerebral tumors in responsiveness to immune checkpoint inhibitor (ICI) treatment and in phenotypes of tumor-associated macrophages (TAMs) in the tumor immune microenvironment (TIME). (A) Schematic illustration of dual CTLA-4/+PD-1 blockade dosing schedule for intracerebral or subcutaneous SB28 tumors. (B) Bioluminescence measurements of SB28 injected intracerebrally and treated with IgG control (gray) or ICI (red). Statistical test: mixed-effects model (n.s.: p>0.05). Error bars: SEM. (C) Tumor volume measurements of SB28 injected subcutaneously and treated with IgG control (gray) or ICI (blue). Statistical test: repeated measures two-wayanalysis of variance (ANOVA; ****p≤0.0001). Error bars: SEM. (D) Volcano plot comparing abundance of tumor-infiltrating leukocyte (TIL) subpopulations in dissociated intracerebral (i.c., red) and subcutaneous (s.c., blue) SB28 tumors using the CyTOF mouse immune cell panel. Statistically significant clusters in volcano plots are highlighted in opaque color and indicated with a cell type label. (E) Biaxial plots of representative raw CyTOF single-cell measurements of CD45, PD-L1, and CD206 on dissociated SB28 subcutaneous or intracerebral tumors. Only CD11b+ events are shown. (F) Mass cytometry data from SB28 subcutaneous or intracerebral tumors were manually gated as shown in (E) on CD11b+ TAMs expressing or lacking PD-L1, CD206, or MHC-II. Frequencies of TAMs expressing all possible permutations of these three markers were quantified. Student’s t-test (***p≤0.001; **p≤0.01; *p≤0.05; n.s.: p>0.05).

Dual CTLA-4/PD-1 blockade was previously reported to prolong survival of GL261 intracerebral tumors, but to be ineffective for SB28.^22^ We hypothesized that SB28 tumors might respond to ICI if grown outside of the immunologically specialized environment of the brain. We therefore compared the effectiveness of dual CTLA-4/PD-1 blockade on SB28 intracerebral tumors versus SB28 tumors grown subcutaneously in the flank (figure 2A). Consistent with the prior report, ICI treatment failed to slow the growth of established intracerebral SB28 tumors (figure 2B). Surprisingly, the same ICI cocktail completely blocked growth of established subcutaneous SB28 tumors in the flank (figure 2C).

We performed mass cytometry of dissociated untreated intracerebral and subcutaneous SB28 tumors to identify populations in the TIME that might contribute to the differential response to treatment in the brain versus the flank. Intracerebral SB28 tumors were distinguished by abundant PD-L1+ myeloid cells (TAMs and microglia) while subcutaneous SB28 tumors trended toward increased T cells and NK cells, and contained significantly more cDC2 cells (CD11c+, CD11b+, CD103−, CD8−, Flt3^int^) which are efficient at priming CD4 T cells^25^ (figure 2D and online supplemental figure S2A).

10.1136/jitc-2020-002181.supp4 Supplementary data

To further explore the myeloid phenotypes present in SB28 tumors grown intracerebrally or subcutaneously, we examined the coexpression of PD-L1, CD206, and MHC-II on tumor-infiltrating CD11b+ myeloid cells. The glioma TAMs in our dataset did not follow a binary split into the classic “M1” proinflammatory and “M2” immunosuppressive phenotypes.^26^ Instead, we observed simultaneous coexpression of M1 markers (MHC-II) and M2 markers (PD-L1, CD206) in every permutation. The immune microenvironment of subcutaneous SB28 tumors was dominated by MHC-II- PD-L1- CD206+ TAMs, while intracerebral SB28 tumors were dominated by MHC-II- PD-L1+ CD206 TAMs (figure 2E, F, online supplemental figure S2B–C). The presence of MHC-II+ TAMs was a defining feature shared by SB28 subcutaneous tumors (figure 2F) and GL261 intracerebral tumors (figure 1D), both of which were responsive to ICI, while MHC-II+ TAMs were essentially absent in the ICI-refractory SB28 intracerebral tumors (figure 2F).

Our data suggest that the lack of response to ICI observed in intracerebral SB28 tumors was not simply a tumor cell-intrinsic property. Rather, ICI-responsiveness could be restored by changing the anatomical tumor site, indicating that tumor cell-extrinsic mechanisms contribute to immune evasion. Single-cell profiling implicated several specific mechanisms, including antigen presentation by DCs and the balance between immunosuppressive and immune-activating TAMs.

### Dual CTLA-4/PD-1 blockade of SB28 subcutaneous tumors elicits systemic immunity, expands cDC2s and is dependent on CD4 T cells and NK cells

**Figure 3:**
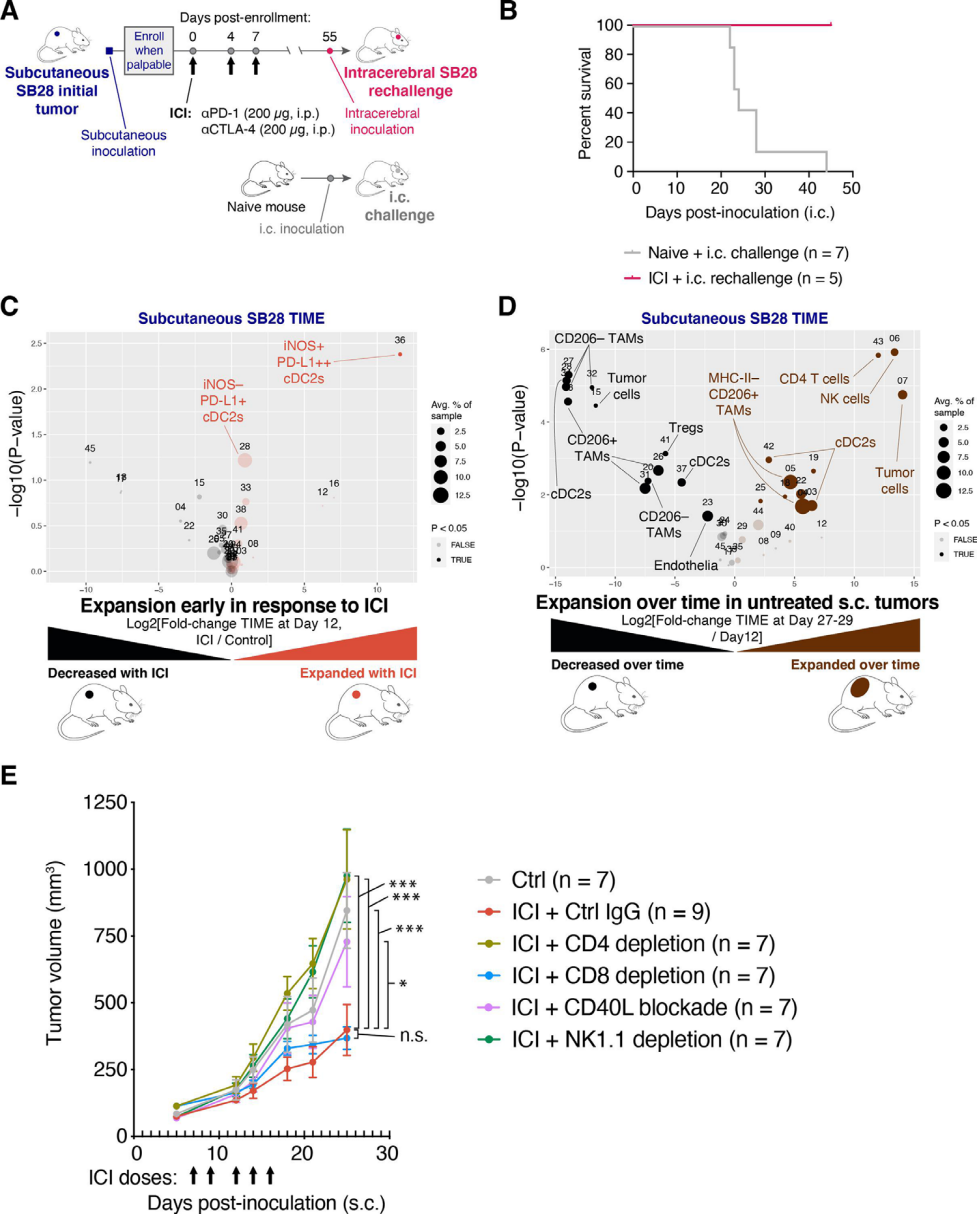
Immune checkpoint inhibitor (ICI) treatment of SB28 subcutaneous tumors can elicit systemic memory and requires similar immune subsets to those involved in natural immune surveillance. (A) Schematic illustration of dosing schedule for SB28 intracerebral tumor rechallenge. (B) Mice previously cured of SB28 subcutaneous tumors (pink line) were rechallenged with SB28 intracerebral tumors. Survival was compared to naive mice (gray line) challenged with SB28 intracerebral tumors and illustrated in a Kaplan-Meier curve. No treatments were administered. (C) Volcano plot comparing abundance of immune subpopulations in dissociated subcutaneous SB28 tumors on day 12 after ICI treatment (orange) versus isotype control treatment (black), using the mouse immune cell panel. Statistically significant clusters in volcano plots are highlighted in opaque color and indicated with a cell type label. (D) Volcano plot as in (C) comparing abundance of immune subpopulations in dissociated subcutaneous SB28 tumors on day 27–29 (brown) versus day 12 (black). (E) Tumor volume measurements of SB28 subcutaneous tumors treated with control IgG (gray) or ICI in the context of either CD4 T cell depletion (light green), CD8 T cell depletion (blue), CD40L blockade (orange), or NK cell depletion (dark green). Depleting or blocking antibodies were administered starting on day −3 or day 0, as described in online supplemental table S6. ICI was administered on days 7, 9, 12, 14, 16 as indicated (arrows). Statistical test: repeated measures two-way analysis of variance (ANOVA; ***p≤0.001; *p≤0.05; n.s.: p>0.05). Error bars: SEM. TAM, tumor-associated macrophage; TIME, tumor immune microenvironment.

To investigate if the ICI-mediated tumor growth inhibition in SB28 subcutaneous tumors resulted in sustained immunological memory, we took mice cured of subcutaneous SB28 tumors and rechallenged them with intracerebral SB28 tumors (figure 3A). All mice previously cured of SB28 subcutaneous tumors rejected the subsequent intracerebral SB28 tumors, while all naive mice died within 45 days of the intracerebral tumor inoculation (figure 3B).

To understand early events in the response to ICI, we performed immune profiling of subcutaneous SB28 tumors resected on day 12 of treatment with either dual CTLA-4/PD-1 or isotype control. The most prominent change was the expansion of a small iNOS+ cDC2 subset in ICI-treated subcutaneous tumors and a trend toward increased iNOS− cDC2s (figure 3C and online supplemental figure 3A).

10.1136/jitc-2020-002181.supp5 Supplementary data

We next compared the TIME of subcutaneous SB28 tumors at early (day 12) versus end stage (day 27–29) time points in untreated mice. The TIME changed dramatically over 2 weeks of unchecked tumor growth. In particular, we observed increases in subsets of cDC2s, NK cells and CD4 T cells at end stage compared with day 12, and concomitant decreases in some subtypes of TAMs and Tregs (figure 3D and online supplemental figure 3B). Notably, NK cells were the most significantly increased subset, representing ~5% of immune cells in end stage tumors, but <0.1% of immune cells in day 12 tumors. To identify the immune populations that mediated the antitumor effect of ICI in subcutaneous tumors, we performed ICI treatment concurrently with depletion of CD4 T cells, CD8 T cells, or NK cells (figure 3E, online supplemental figure S3C). We also tested a blocking antibody against CD40 ligand (CD40L; CD154) to disrupt licensing of DCs.^27 28^ The efficacy of ICI in subcutaneous SB28 tumors was strongly dependent on CD4+ T cells, NK cells and CD40:CD40L interactions. There was not an absolute requirement for CD8+ T cells, but tumor regression in response to ICI was seen in 0/8 of the CD8-depleted mice, compared with 3/9 of the non-depleted mice, suggesting that CD8 T cells played a minor role in ICI-mediated tumor rejection (online supplemental figure S3D).

These data suggest that ICI can elicit successful priming against SB28 tumor antigens in an extracranial setting, and that tumor control in that context is a multi-step process involving NK cells, DCs, CD4 T cells, and to a lesser extent, CD8 T cells.

### Systemic FLT3L expands cDC2s and pDCs in the SB28-OVA-FL intracerebral model

Results in the subcutaneous SB28-parental tumor model suggested that cDC2s play a critical role in establishing antitumor immunity, especially by priming CD4 T cells. Major DC subsets in tumors and tumor-draining lymph nodes can include: (1) plasmacytoid DCs (pDCs) that produce type I IFN in response to viral infection; (2) cDC1s that cross-present MHC-I epitopes to CD8 T cells; and (3) cDC2s that mediate priming of CD4 T cells via MHC-II.^25^ We hypothesized that increasing DC abundance at the intracerebral site might restore antitumor immunity. We therefore investigated if treatment with FLT3L, a key growth factor for DCs, would boost antigen presentation and T cell priming.

**Figure 4:**
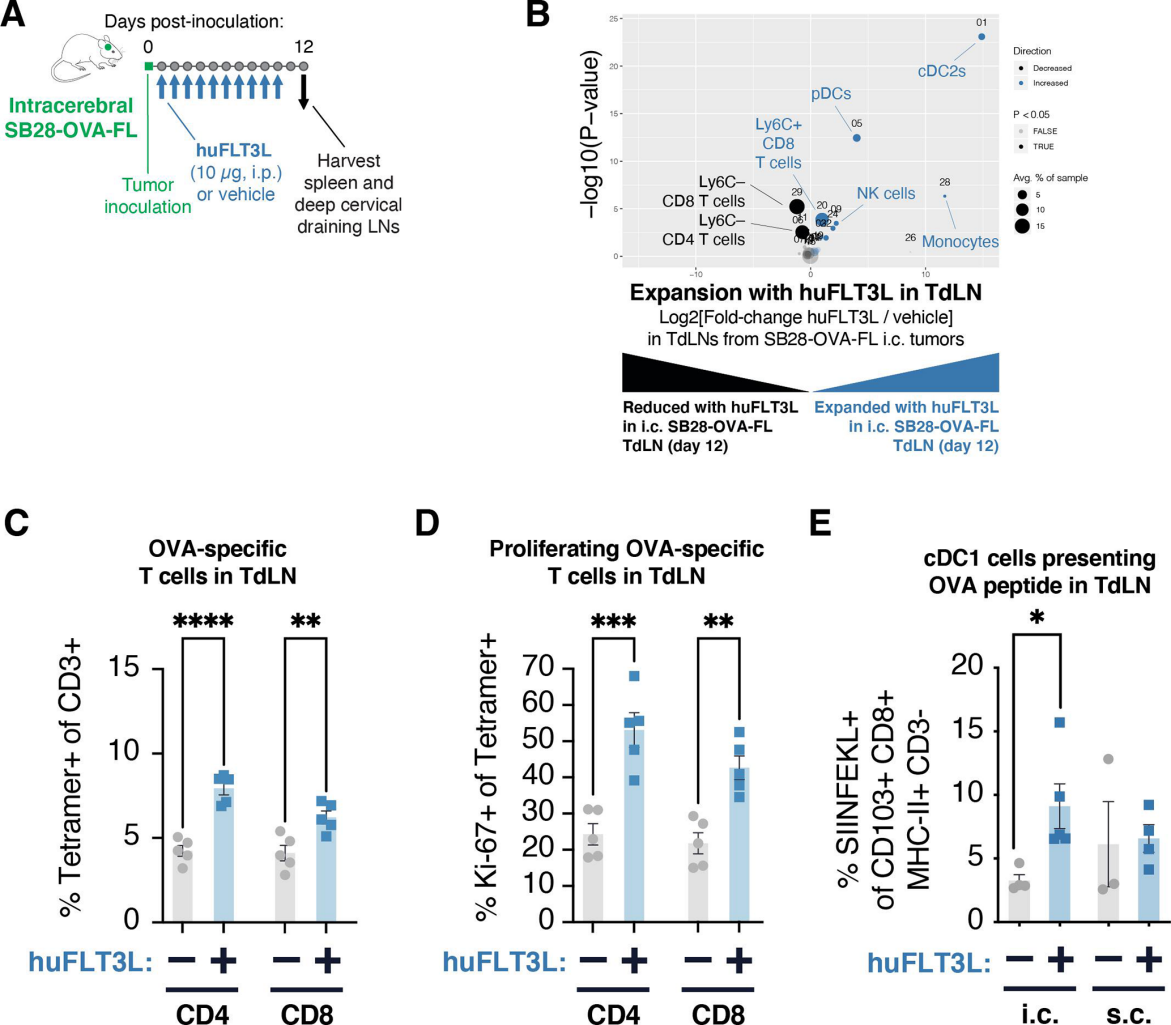
Treatment with FLT3L expands multiple dendritic cell subsets and improves antigen presentation in intracerebral SB28-OVA-FL tumors. (A) Schematic illustration of dosing schedule (daily for 10 days) for SB28 intracerebral huFLT3L-Fc treatment and analysis of tumor-draining lymph nodes (TdLNs) and tumors at day 11–12 post-tumor injection. (B) Treatment with huFLT3L expands cDC2s, pDCs, and Ly6C+ CD8 T cells in TdLNs of mice with SB28-OVA-FL tumors at day 12 post-inoculation. Volcano plot comparing abundance of immune subpopulations in TdLNs from huFLT3L-treated (blue) and control-treated (black) intracerebral SB28-OVA-FL. Statistically significant clusters in volcano plots are highlighted in opaque color and indicated with a cell type label. (C) Frequency of OVA tetramer+ CD4 and CD8 T cells (out of CD3+ T cells) from TdLNs at day 12 post-inoculation of SB28-OVA-FL tumors treated with control IgG or huFLT3L-Fc. (D) Frequency of proliferating (Ki-67+) T cells among the OVA tetramer+ cells in (C). (E) Treatment with huFLT3L improves presentation of a tumor-derived antigen. Frequency of cDC1 cells that were presenting SIINFEKL-peptide on MHC class I were quantified by flow cytometry with an anti-SIINFEKL-MHC-complex antibody. Same samples as online supplemental figure S4J.

To explore tumor antigen presentation in more detail, we used SB28-OVA-FL, a variant of SB28 that was engineered to express the full-length chicken ovalbumin (OVA) gene. We inoculated mice either intracerebrally or subcutaneously with SB28-OVA-FL, and administered soluble half-life-extended (Fc-fused) human FLT3L (huFLT3L) daily for 10 days (figure 4A). The systemic huFLT3L treatment caused expansion of cDC2s and pDCs (figure 4B and online supplemental figure S4A) in draining lymph nodes and spleens (online supplemental figure S4B, C) at day 12. The expansion in draining lymph nodes was particularly dramatic, with pDCs increasing from 0.4% to 5.4%, and cDC2 expanding from 0% to 4.2%. The expansion of DC subsets was accompanied by upregulation of Ly6C+ on CD8 T cells. Induction of Ly6C on T cells is primarily driven by IFN-α signaling,^29^ which may indicate that the expanded DCs were secreting type I IFNs.

10.1136/jitc-2020-002181.supp6 Supplementary data

### Systemic FLT3L treatment improves priming and expansion of tumor-specific CD4 T cells

To explore the consequences of FLT3L treatment on T cell priming and expansion, we stained for OVA-specific CD4 and CD8 T cells using MHC-tetramers at day 12 after intracerebral SB28-OVA injection. There was an expansion of OVA-specific CD4 but not CD8 T cells in the tumor-draining lymph nodes (figure 4C) and spleen (online supplemental figure S4D) with huFLT3L treatment. Moreover, huFLT3L treatment was associated with significantly increased proliferation (Ki-67+ staining) of OVA-specific CD4 and CD8 T cells in tumor-draining lymph nodes (figure 4D) and spleen (online supplemental figure S4E). These T cells were not restricted to the lymph node. In SB28-OVA intracerebral tumors harvested on day 12, we detected OVA-specific CD4 and CD8 T cells, including Ki-67+ cells, suggesting proliferation and trafficking to the tumor (online supplemental figure S4F–I).

We next asked if cross-presenting cDC1s (CD103+ CD8+) were involved in the priming process in the tumor-draining lymph node. These cells have a unique capability to prime CD8 T cells against MHC class I-restricted tumor antigens and have been shown to be essential for the effect of ICI in some tumor models.^30 31^ To assess the presentation of tumor antigen on the cell surface of cDC1s, we used a fluorescent antibody that recognizes the OVA SIINFEKL peptide docked in MHC-I. huFLT3L treatment robustly expanded the cDC1 population in intracerebral SB28-OVA-FL tumor-draining lymph nodes and the fraction of these cross-presenting SIINFEKL, but it had no significant effect on cDC1 abundance or cross-presentation in subcutaneous tumor-draining lymph nodes (figure 4G and online supplemental figure S4J, K).

Taken together, these results show that huFLT3L treatment induced expansion of cDC2s, pDCs, and tumor antigen-specific CD4 T cells, while also enhancing tumor antigen cross-presentation on cDC1s, and proliferation of tumor antigen-specific CD8 T cells

### huFLT3L monotherapy extends survival in the SB28 intracerebral model but does not enhance response to ICI

**Figure 5:**
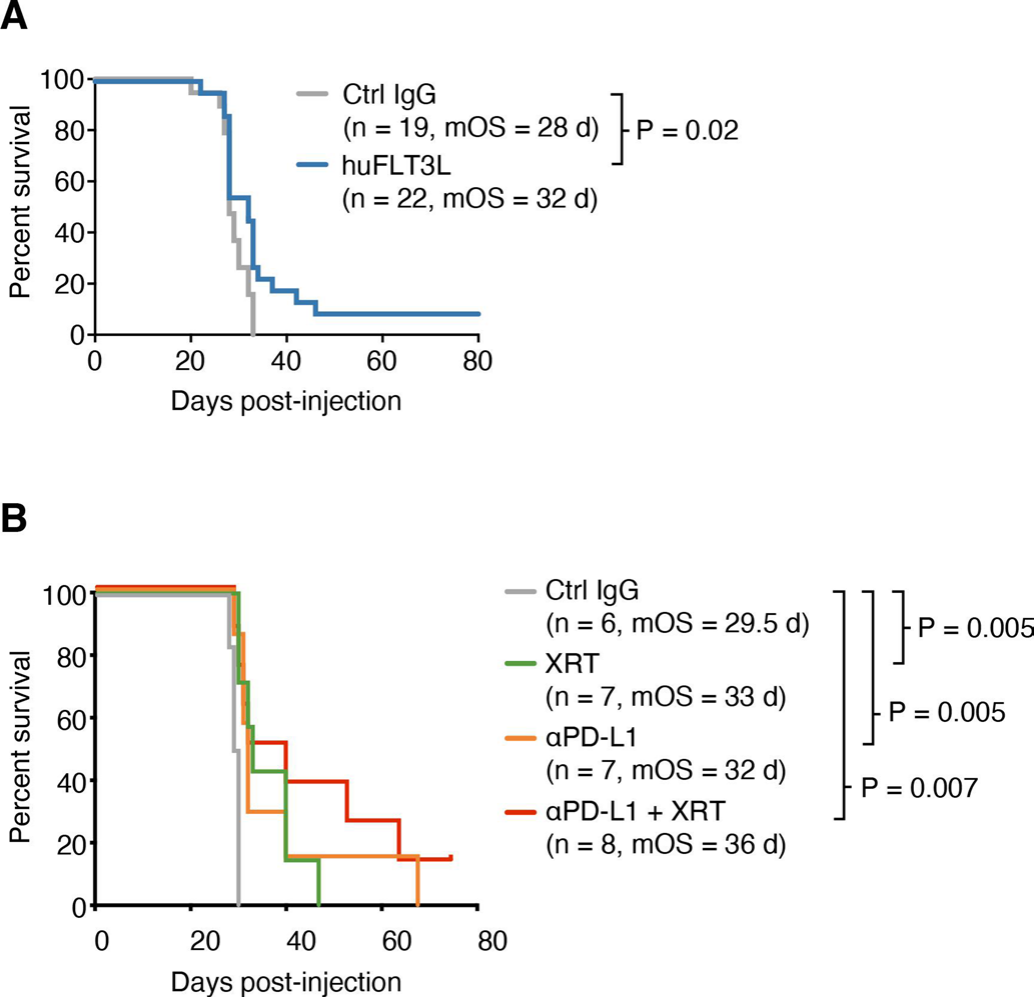
Improved antigen presentation combined with checkpoint blockade extends survival of mice with SB28 intracerebral tumors. (A) Kaplan-Meier curves of intracerebral SB28 treated with IgG control (gray) or huFLT3L (blue). Log-rank test p values and median overall survival (mOS) are shown. Data from two experiments were aggregated. (B) Kaplan-Meier curves of intracerebral SB28 treated with IgG control (same as (B), gray), radiation (XRT, same as (B), green), anti-PD-L1 (yellow) or XRT and ant-PD-L1 (blue). Log-rank test p values and mOS are shown.

We asked whether huFLT3L would improve survival in mice bearing intracerebral SB28 (parental) tumors which do not respond to dual CTLA-4/PD-1 blockade. All mice treated with an isotype control IgG died within 33 days of inoculation, while 5 of 22 mice (23%) receiving huFLT3L monotherapy lived more than 35 days (figure 5A). Two of the huFLT3L-treated mice were effectively cured, surviving more than 80 days. We postulated that radiation (XRT)—a cornerstone of GBM clinical care—would promote priming by releasing tumor antigens for subsequently presentation by DCs. Therefore, we tested huFLT3L and fractionated high dose radiation therapy (XRT; 8 Gy×3 doses), alone and in combination. The combination of huFLT3L+XRT improved survival relative to control-treated mice, but did not provide significant additional benefit over XRT or huFLT3L alone (online supplemental figure S5A). Paradoxically, combining huFLT3L treatment with dual CTLA-4/PD-1 blockade trended toward shorter survival (online supplemental figure S5B). Altogether, these results support a small therapeutic benefit of huFLT3L treatment in SB28 intracerebral tumors.

10.1136/jitc-2020-002181.supp7 Supplementary data

### PD-L1 blockade extends survival in the SB28 intracerebral model

Our immune profiling of patient samples and mouse models indicated an overabundance of PD-L1+ TAMs in human GBM and SB28 intracerebral tumors. While dual CTLA-4/PD-1 blockade was ineffective in SB28 intracerebral tumors (figure 2B), PD-L1 blockade slightly extended median survival relative to isotype control IgG-treated tumors (32 vs 29.5 days, p=0.005) (figure 5B). This effect was further enhanced by addition of XRT (36 vs 29.5 days, p=0.007). Addition of huFLT3L (eg, triple-combination therapy) did not improve survival relative to PD-L1+ XRT (online supplemental figure S5C). We next sought to enhance the maturation or activation of DCs. Addition of a CD40-agonistic antibody to huFLT3L treatment trended toward providing additional benefit, but did not significantly improve survival relative to huFLT3L alone (online supplemental figure S5D). PolyIC:LC did not provide any additional benefit relative to huFLT3L alone (online supplemental figure S5D).

### huFLT3L in combination with immunotherapy modulates DCs and T cells in the SB28 intracerebral model

**Figure 6:**
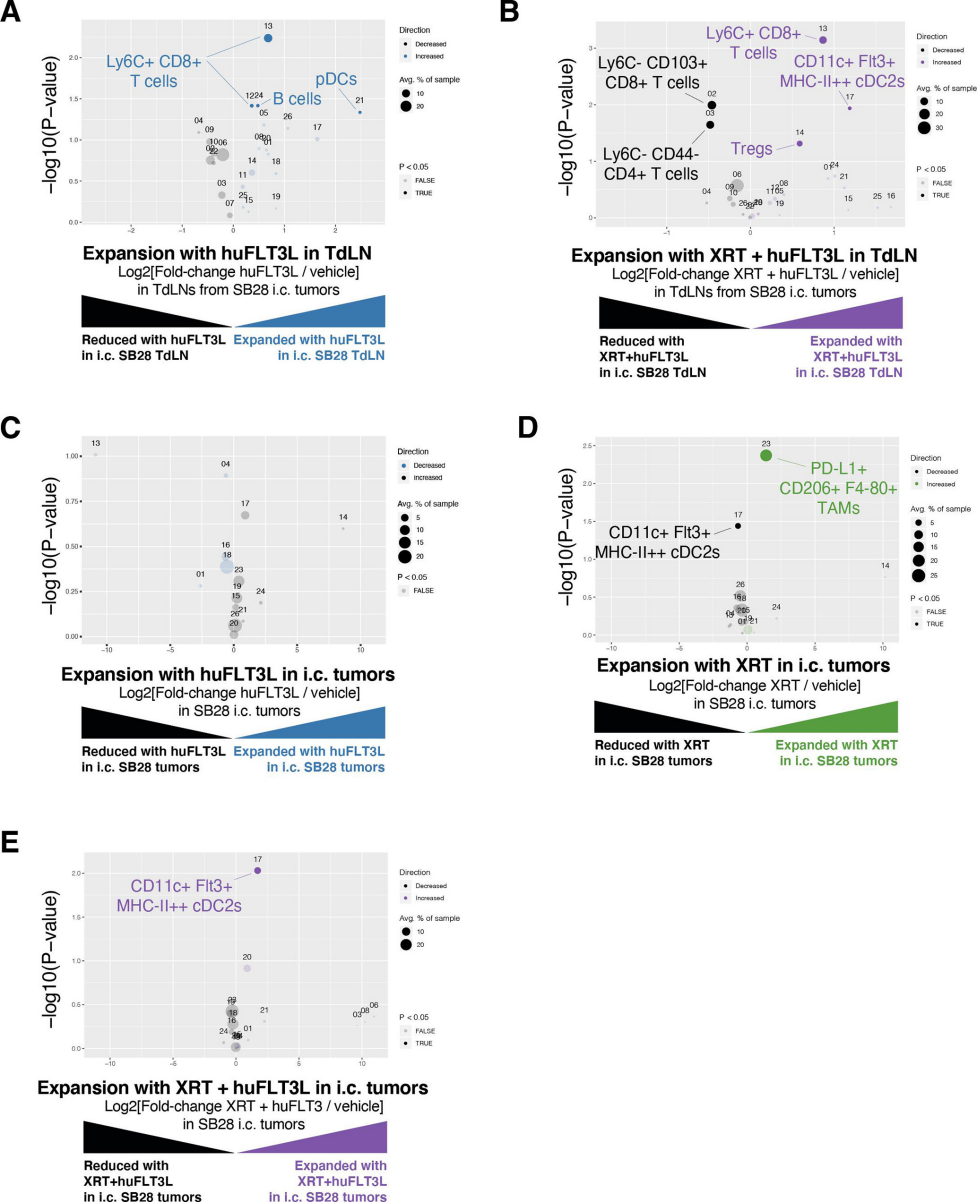
huFLT3L in combination with immunotherapy modulates dendritic cells (DCs) and T cells in the SB28 intracerebral model. (A) Volcano plot comparing abundance of immune subpopulations in tumor-draining lymph nodes (TdLNs) from intracerebral SB28 treated with huFLT3L (blue) versus control (black) using mass cytometry. Statistically significant clusters in volcano plots are highlighted in opaque color and indicated with a cell type label. (B) Volcano plot as in (A) comparing radiation (XRT)+huFLT3L (purple) versus vehicle-treated (black) in TdLNs. (C) Volcano plot comparing abundance of immune subpopulations in end stage tumors from intracerebral SB28 treated with huFLT3L (blue) versus vehicle-treated (black) using CyTOF. No statistically significant clusters were detected. (D) A single dose of radiation (10 Gy) induced PD-L1+ CD206+ (M2), tumor-associated macrophages and reduced DCs in SB28 i.c. tumors at endpoint. Volcano plot as in (C) comparing radiation therapy (XRT) (green) versus vehicle-treated (black). (E) Radiation (1×10 Gy)+FLT3L restored DCs in SB28 i.c. tumors at endpoint, compared to XRT alone. Volcano plot as in (C) comparing XRT+huFLT3L (purple) versus vehicle-treated (black).

To explore the consequences of therapeutic intervention on the TIME, we performed mass cytometry of tumors and tumor-draining lymph nodes at end stage. huFLT3L monotherapy was associated with expansion of pDCs and Ly6C+ CD8+ T cells in tumor-draining lymph nodes at end stage, but there was no change in total CD8+ T cell numbers (figure 6A and online supplemental figure S6A). The dual therapy of XRT+hFLT3L had similar effects on tumor-draining lymph nodes, but produced fewer pDCs and more cDC2s. This combination therapy also caused a 1.5-fold expansion of Tregs coupled with a 1.5-fold reduction of naive (CD44−) CD4 T cells (figure 6B and online supplemental figure S6A). Similar increases in Tregs were observed when huFLT3L was combined with dual CTLA-4/PD-1 blockade (online supplemental figure S6B, C). Taken together, these results suggest that FLT3L combination immunotherapies can promote a proinflammatory microenvironment in draining lymph nodes, at the cost of a compensatory expansion of Tregs that may ultimately contribute to immune evasion.

10.1136/jitc-2020-002181.supp8 Supplementary data

As for the immune compartment within the end stage tumors, FLT3L treatment had no effect on any CD45+ cell population (figure 6C and online supplemental figure S6D). XRT monotherapy reduced cDC2s and expanded a PD-L1+ CD206+ TAM population, both of which would be undesirable changes for antitumor immunity (figure 6D and online supplemental figure S6E, online supplemental figure S7A). Combining huFLT3L treatment with XRT reversed these effects and increased the cDC2 population again, potentially creating a tumor microenvironment more supportive of T-cell priming (figure 6E and online supplemental figure S6F). Neither XRT monotherapy, huFLT3L monotherapy, nor the combination altered the abundance of intratumoral T cells at endpoint (figure 6C–E).

10.1136/jitc-2020-002181.supp9 Supplementary data

We then investigated whether these treatments cause a change in surface marker profiles on intratumoral T cells. Normal human brain contains rare CD69-high CD8+ T cells, a type of tissue-resident memory cells (T_RM_).^32^ We also observed that the CD8+ T cells in untreated tumors were CD69-high CCR7-low PD-1-high (online supplemental figure S6G). Both XRT and huFLT3L monotherapies, as well as the combination, promoted a shift in the phenotype of intratumoral CD8+ T cells characterized by downregulation of PD-1 and CD69 and upregulation of CCR7 (online supplemental figure S6G). This change in T cell surface marker expression, especially the upregulation of CCR7, may suggest differentiation of T_RM_ cells toward a central memory T cell fate.

In summary, FLT3L treatment, blockade of PD-L1 and radiation all improved survival in SB28 intracerebral tumors. There was a benefit of combining PD-L1 blockade with radiation, but triple combinations did not provide additional survival benefit. Each treatment or combination impacted the immune microenvironment in the tumor and draining lymph node in slightly different ways, primarily affecting T cell and DC subsets. Our data suggest that that DCs and PD-L1+ TAMs are malleable cell populations in human and mouse gliomas that can be modulated therapeutically using huFLT3L, anti-PD-L1 and radiation.

## Discussion

Recently, several groups have used mass cytometry to profile the TIME of GBM in patients and in mouse models. These studies have shown abundant myeloid cells in the TIME of GBM as well as brain metastases of melanomas or carcinomas,^9 10 17 33 34^ and that primary brain tumors such as glioma are especially enriched in myeloid cells and poor in DCs.^9 10^ Our data agree with a recent study showing that immunosuppressive macrophages (PD-L1+ CD73+) are enriched in patients with GBM.^17^ Immune profiling studies from Lathia and colleagues have focused on myeloid-derived suppressor cells (MDSCs) as a dynamic cell population both in the GBM patients’ blood and the TIME. They showed that the abundance of MDSCs can have prognostic value and that it can be modified by metronomic gemcitabine treatment, thereby also affecting other immune cell populations.^34 35^ Furthermore, a comparative mass cytometry study analyzing five syngeneic mouse models of GBM (including GL261 but not SB28) showed prominent macrophage infiltration.^36^ Interestingly, all five mouse models had significantly more infiltration of DCs as compared with human patients with GBM, suggesting that SB28 could be an important model to mimic the deficiency of DC recruitment seen in human GBM. Further profiling efforts in mice and humans will clarify which models recapitulate specific aspects of the human GBM TIME most faithfully, as none of the models are likely to model all aspects of human disease.

Our study suggests that intracerebral GBM tumors fail to elicit antitumor immunity due to defects early in the cancer-immunity cycle,^37^ specifically, poor priming of antigen-specific T-cells at the tumor-draining lymph node. Additionally, there may be defects later in the cycle mediated by PD-L1+ cells in the TIME, as suggested by the efficacy of PD-L1 blockade. Although PD-1 and PD-L1 checkpoint blockade therapies disrupt the same signaling axis, our results indicate that PD-L1 blockade is more effective than dual CTLA-4/PD-1 blockade in the SB28 model. This is in line with a recent report of a modest positive effect in interim data from a phase II clinical trial combining XRT and PD-L1 blockade in unmethylated GBM.^38^ In a previous study of the GBM microenvironment by flow cytometry, myeloid cells were proposed as the dominant source of PD-L1.^39^ Consistent with these results, our mass cytometry profiling showed that PD-L1-expressing myeloid cells were abundant in human GBM as well as the SB28 model. It has been shown that SB28 glioma cells do not express PD-L1 under basal conditions, but PD-L1 expression can be induced in vitro by IFN-g.^22^ Why anti-PD-L1 was more effective than dual CTLA-4/PD-1 blockade is unknown, but there are several possible mechanisms that distinguish these therapies. Anti-PD-L1 is able to disrupt the *cis* interaction between PD-L1 and B7-1 (CD80) on DCs, allowing CD80 to activate T cells via CD28.^40^ Anti-PD-L1 is also able to act directly on tumor cells, driving cytokine production and in vivo phagocytic activity of glioma TAMs in some contexts.^41^ Finally, dual CTLA-4/PD-1 blockade can induce apoptosis of tumor-specific T cells in preclinical models with low tumor burden.^42^

Several lines of evidence suggest that defective antigen presentation is of central importance in explaining the non-responsiveness of GBM to ICI. Our results in the SB28 model agree with recent reports that brain tumors are poorly infiltrated by DCs.^9 10^ We observed dramatic differences in antigen presentation and responsiveness to immunotherapy when SB28 tumors were grown in the flank as opposed to the brain. SB28 flank tumors showed a significant influx of cDC2s into the tumors and cDC1s into the tumor-draining lymph nodes, as compared with SB28 intracerebral tumors. This influx was further increased by dual CTLA-4/PD-1 blockade. Further supporting a central role for DCs, the dual CTLA-4/PD-1 blockade-mediated inhibition of SB28 flank tumors was dependent on CD40 signaling, which is important for licensing of DCs. Treatment with huFLT3L increased the frequency of OVA-presenting cDC1s in cervical lymph nodes and modestly improved survival, thus highlighting the fundamental defects in antigen presentation in the brain, and providing a rationale for FLT3L-based strategies to overcome this challenge. This is also in line with a recent study in which dual CTLA-4/PD-1 blockade promoted rejection of melanoma brain metastases only when an extracranial melanoma tumor was present as well.^43^ Another recent report showed that intracerebral delivery of VEGF-C can boost antigen trafficking to deep cervical lymph nodes and drive rejection of GL261 tumors.^44^ This is a promising new way to modulate antigen trafficking and an exciting candidate for follow-up in the SB28 model in combination with the approaches tested here.

The dual CTLA-4/PD-1-mediated rejection of SB28 flank tumors was dependent on CD4 T cells and NK cells, while CD8 T cells were less important, suggesting an unconventional effector mechanism of antitumor immunity. In our hands, SB28 tumor cells did not express MHC-II, consistent with previous reports,^22^ so it is unlikely that effector CD4 T cells are directly mediating tumor rejection in this model. Based on our depletion data, the critical effector cells in ICI-treated SB28 subcutaneous tumors are likely NK cells that are potentiated by antigen-specific CD4 T cells. NK cells and CD4 T cells were also expanded over time in untreated subcutaneous SB28 tumors, indicating they are involved in tumor surveillance. The essentiality of CD4 and NK cells in the SB28 model is particularly interesting in light of three other findings: (1) priming in the periphery elicited systemic immunity, (2) the efficacy of dual CTLA-4/PD-1 blockade in the flank required CD40-mediated licensing of DCs, and (3) cDC2 cells were abundant even in untreated SB28 flank tumors. Integrating these pieces of evidence, we propose a model of the systemic antitumor immunity that we observed in SB28 flank tumors treated with dual CTLA-4/PD-1 blockade: tumor antigen is processed and presented by cDC2s early in the life of the tumor, but it is not sufficient to activate CD4 T cells due to negative signals from PD-1 and CTLA-4. The addition of dual CTLA-4/PD-1 checkpoint blockade expands cDC2s and lowers the threshold of activation, promoting CD4 T cell expansion and release of proinflammatory cytokines (eg, interleukin (IL)-2, IFN-γ, IL-12). These cytokines, in turn, promote expansion and activation of NK cells,^45^ which act as the main effector cell population to mediate SB28 flank tumor rejection. We postulate that memory CD4 T cells are also key mediators of systemic immunity in this model, protecting mice from intracerebral rechallenge by secreting proinflammatory cytokines and activating NK cells at the new tumor site. The development of protective immune memory may or may not require ICI, but this could not be tested in the current experimental setup because ICI treatment was necessary to achieve rejection of the subcutaneous tumors prior to rechallenge with intracerebral tumors.

In SB28 intracerebral tumors, FLT3L treatment led to a modest but significant increase in overall survival, including sustained remissions of over 60 days in a subset of mice. However, combining FLT3L with other agents or radiation did not lead to synergistic or additive effects. To harness the potential of FLT3L treatment we need to understand what distinguishes the few cured mice from the non-responders and why combination of dual CTLA-4/PD-1 blockade with FLT3L appeared to negate the benefits of FLT3L treatment. Evidence from our mass cytometry profiling points to regulatory T cells as a key player in this system. We observed that FLT3L monotherapy caused expansion of pDC and Tregs in tumor-draining lymph nodes, which was further enhanced when FLT3L was combined with XRT or dual CTLA-4/PD-1 blockade. The mechanism underlying this expansion of Tregs may be similar to that previously described in human breast and ovarian cancer^46 47^ and the B16 mouse model of melanoma.^30^ In those studies, tolerogenic pDCs engaged ICOS on CD4 T cells, thereby promoting differentiation to Tregs, and causing immunosuppression in tumors. Similarly, expansion of DCs with FLT3L in the SB28 intracerebral model may initiate homeostatic feedback mechanisms that blunt the efficacy of FLT3L as a monotherapy or in combination with ICI.

Based on our results here, FLT3L shows potential as a therapeutic strategy to improve antigen presentation in GBM, but combination strategies will likely be necessary to achieve a clinical benefit. Human clinical trials using FLT3L in mesothelioma and some carcinomas have reported limited effect as a single agent.^48 49^ However, preclinical studies in several mouse tumor models suggest that FLT3L can be effective, particularly when used in combination with other agents. In a mouse model of pancreatic adenocarcinoma, a combination of FLT3L, XRT and CD40 agonist was used to skew the DC phenotype away from a Th2 and Th17 response toward a Th1 response.^50^ In a mouse model of BRAF-mutant melanoma, CD103+ cDC1s were essential for an effective anti-PD-L1 response and could be expanded by FLT3L. However, FLT3L treatment also led to proliferation of immature progenitor DCs with the capacity to expand Tregs. Combining FLT3L with an agent to promote maturation of DCs (poly I:C) was essential to promote an effective anti-PD-L1 mediated immune response.^30^ These examples were all done in extracranial tumor models, but in the intracerebral SB28 model, the addition of polyIC:LC or CD40 agonist did not substantially improve FLT3L therapy.

Overall, this work illustrates how tumor location, antigenicity, and immune microenvironment can influence the efficacy of immunotherapies. It underscores how these factors must work in harmony to tip the scales toward effective antigen presentation and antitumor immunity. Our data demonstrate that the main hindrance for effective immunotherapy of glioma is poor antigen presentation, rather than intrinsic immunosuppressive properties of the glioma cells themselves. Accurate syngeneic tumor models and deep immune profiling should be leveraged to further elucidate the immunosuppressive mechanisms that are unique to brain tumors, and to guide the rational combination of immunotherapies to control these challenging tumors.

10.1136/jitc-2020-002181.supp10 Supplementary data

10.1136/jitc-2020-002181.supp11 Supplementary data

## Data Availability

Processed mass cytometry data with PhenoGraph cluster assignments will be made available upon publication at FlowRepository.org (identifiers FR-FCM-Z3HK, FR-FCM-Z3HL, FR-FCM-Z3HM, FR-FCM-Z3HN, and FR-FCM-Z3HX). Mass cytometry analysis scripts will be made available on GitHub upon publication at https://github.com/esimonds/PhenoSOM. Details and accession numbers are listed in the Online Supplemental Methods.
