# Supplementary material for: Deep immune profiling reveals targetable mechanisms of immune evasion in immune checkpoint inhibitor-refractory glioblastoma
Source: J Immunother Cancer. 2021 Jun 2;9(6):e002181. doi: 10.1136/jitc-2020-002181 (PMC8183210; doi:10.1136/jitc-2020-002181)
Supplement: Supplementary data [file jitc-2020-002181supp002.pdf]

Table S1 – Patient Details

Patient ID	Year of collection	Patient sex	Patient age	Diagnosis	Tissue type	EGFR gene amplification
SF10711	2016	M	58	Astro Grade II	Primary tumor biopsy	Not tested
SF10941	2016	M	48	GBM	Primary tumor biopsy	Yes
SF10953	2016	M	37	GBM	Primary tumor biopsy	No
SF10985	2016	M	38	Adult GBM	Primary tumor biopsy	No
SF10986	2016	F	52	GBM	Primary tumor biopsy	No
SF11048	2016	M	71	GBM	Primary tumor biopsy	Yes
SF11071	2016	M	66	GBM	Primary tumor biopsy	Yes
SF11159	2017	M	60	GBM	Primary tumor biopsy	No
SF11232	2017	M	61	GBM	Primary tumor biopsy	No
SF11318A	2017	M	55	GBM	Primary tumor biopsy	No
SF11331	2017	M	55	GBM	Primary tumor biopsy	No
SF11378	2017	M	53	GBM	Primary tumor biopsy	Not tested
SF11447	2017	M	60	HGG	Primary tumor biopsy	No
SF11496	2017	M	43	GBM	Primary tumor biopsy	No
SF11503	2017	M	76	GBM	Primary tumor biopsy	No
SF11512	2017	M	29	GBM	Primary tumor biopsy	No
SF11517	2017	F	54	GBM	Primary tumor biopsy	Yes
SF11723	2018	F	27	GBM	Primary tumor biopsy	No
COH001	2017	M	16	GBM	Primary tumor biopsy	Not tested
IPIKID-97 T1	2017	F	44	Kidney cancer	Primary tumor biopsy	Not tested
CIPSOCKID-01 T1	NA	NA	NA	Kidney cancer	Primary tumor biopsy	Not tested
IPIKID-103	2017	M	65	Kidney cancer	Primary tumor biopsy	Not tested
IPIKID-073	2017	M	60	Kidney cancer	Primary tumor biopsy	Not tested
IPIKID-111 T1	2017	M	27	Kidney cancer	Primary tumor biopsy	Not tested
IPIKID-076	NA	NA	NA	Kidney cancer	Primary tumor biopsy	Not tested
IPIKID-109 T1	2017	M	84	Kidney cancer	Primary tumor biopsy	Not tested
CIPSOCKID-05 T1	2018	NA	NA	Kidney cancer	Primary tumor biopsy	Not tested
CIPSOCKID-06 T1	2018	NA	NA	Kidney cancer	Primary tumor biopsy	Not tested
IPIKID-100	2017	F	79	Kidney cancer	Primary tumor biopsy	Not tested
IPIKID-101	NA	NA	NA	Kidney cancer	Primary tumor biopsy	Not tested
CIPSOCKID-010 N1	2018	NA	NA	Kidney cancer	Normal adjacent tissue	Not tested
CIPSOCKID-010 T1	2018	NA	NA	Kidney cancer	Primary tumor biopsy	Not tested
CIPSOCKID-010 M1	2018	NA	NA	Kidney cancer	Metastatic tumor biopsy	Not tested
IPISRC-09	NA	NA	NA	Sarcoma	Primary tumor biopsy	Not tested
IPISRC-013 N1	NA	NA	NA	Sarcoma	Normal adjacent tissue	Not tested
IPISRC-013 T1	NA	NA	NA	Sarcoma	Primary tumor biopsy	Not tested
IPISRC-016	2017	NA	NA	Sarcoma	Primary tumor biopsy	Not tested
IPISRC-10	NA	NA	NA	Sarcoma	Primary tumor biopsy	Not tested

NA: Data not available

Table S2 – Human T Cell CyTOF Antibody Panel

Channel	Marker	Staining step	Manufacturer	Clone	Staining Concentration (µg/mL)	Used for clustering	Notes
89	CD45	After Fc block	Fluidigm	HI30	--	Yes	
102	Cell-ID 20-plex Pd barcode	After thaw	Fluidigm	--	--	No	
104	Cell-ID 20-plex Pd barcode	After thaw	Fluidigm	--	--	No	
105	Cell-ID 20-plex Pd barcode	After thaw	Fluidigm	--	--	No	
106	Cell-ID 20-plex Pd barcode	After thaw	Fluidigm	--	--	No	
108	Cell-ID 20-plex Pd barcode	After thaw	Fluidigm	--	--	No	
110	Cell-ID 20-plex Pd barcode	After thaw	Fluidigm	--	--	No	
113	CD235ab	After Fc block	BioLegend	HIR2	1	Yes	Two antibodies on same channel as dump for platelets/erythrocytes
113	CD61	After Fc block	BioLegend	C1-PL2	1	Yes	Two antibodies on same channel as dump for platelets/erythrocytes
115	CD44	After Fc block	BioLegend	IM77	1	Yes	
139	Unused	--	--	--	--	Yes	Empty channel
140	CD66	After Fc block	BD Biosciences	b1.1	3	Yes	
141	CD16	After Fc block	BioLegend	3G8	1	Yes	
142	TCR Vd2	After Fc block	BioLegend	B6	2	Yes	
143	CD45RA	After Fc block	Fluidigm	HI100	(1:100)	Yes	
144	CTLA-4	After Perm-S	eBioscience	14D3	1	Yes	
145	CD4	After Fc block	Fluidigm	RPA-T4	(1:100)	Yes	
146	CD8a	After Fc block	Fluidigm	RPA-T8	(1:100)	Yes	
147	CD11c	After Fc block	Fluidigm	Bu15	(1:100)	Yes	
148	ICOS	After Fc block	BioLegend	C398.4A	2	Yes	
149	CD45RO	After Fc block	Fluidigm	UCHL1	(1:100)	Yes	
150	LAG-3	After Fc block	Fluidigm	874501	(1:100)	Yes	
151	CD14	After Fc block	Fluidigm	M5E2	(1:100)	Yes	
152	TCRγδ	After Fc block	BioLegend	B1	3	Yes	
153	TIM-3	After Fc block	Fluidigm	F38-2E2	(1:100)	Yes	
154	TIGIT	After Fc block	Fluidigm	MBSA43	(1:100)	Yes	
155	PD-1	After Fc block	Fluidigm	EH12.2H7	(1:100)	Yes	
156	Vimentin	After Fc block	Fluidigm	RV202	(1:100)	Yes	
157	Epcam	After Fc block	BioLegend	9C4	1	Yes	
158	CD135 (OX40)	After Fc block	Fluidigm	ACT35	(1:100)	Yes	
159	CD137 (4-1BB)	After Fc block	BioLegend	4B4-1	2	Yes	
160	EOMES	After Perm-S	eBioscience	WD1928	2	Yes	
161	T-bet	After Perm-S	Fluidigm	4B10	(1:100)	Yes	
162	FoxP3	After Perm-S	Fluidigm	PCH101	(1:100)	Yes	
163	BTLA	After Fc block	Fluidigm	MIH26	(1:100)	Yes	
164	CD161 (KLRB1)	After Fc block	Fluidigm	HP-3G10	(1:100)	Yes	
165	VISTA	After Fc block	R&D Systems	730804	4	Yes	
166	NKG2D	After Fc block	Fluidigm	ON72	(1:100)	Yes	
167	CD27	After Fc block	Fluidigm	L128	(1:100)	Yes	
168	CD127 (IL-7Ra)	After Fc block	Fluidigm	A019D5	(1:100)	Yes	
169	CD25	After Fc block	Fluidigm	2A3	(1:100)	Yes	
170	CD3	After Fc block	Fluidigm	UCHT1	(1:100)	Yes	
171	GITR	After Fc block	BioLegend	621	8	Yes	
172	Ki-67	After Perm-S	Fluidigm	B56	(1:100)	Yes	
173	Granzyme B	After Perm-S	ThermoFisher	CLB-B11	1	Yes	
174	HLA-DR	After Fc block	Fluidigm	L243	(1:100)	Yes	
175	PD-L1	After Fc block	Fluidigm	29E.2A3	(1:100)	Yes	
176	CD56	After Fc block	Fluidigm	NCAM16.1	(1:100)	Yes	
191	DNA	Final step	Fluidigm	--	--	No	Ir-intercalator
193	DNA	Final step	Fluidigm	--	--	No	Ir-intercalator
194	Viability	Before PFA	Fluidigm	--	--	No	Cisplatin
195	Viability	Before PFA	Fluidigm	--	--	No	Cisplatin
198	Viability	Before PFA	Fluidigm	--	--	No	Cisplatin
209	CD11b	After Fc block	Fluidigm	ICRF44	(1:100)	Yes	

Table S3 – Human Myeloid CyTOF Antibody Panel

Channel	Marker	Staining step	Manufacturer	Clone	Staining Concentration (µg/mL)	Used for clustering	Notes
89	CD45	After Fc block	Fluidigm	HI30	--	Yes	
102	Cell-ID 20-plex Pd barcode	After thaw	Fluidigm	--	--	No	
104	Cell-ID 20-plex Pd barcode	After thaw	Fluidigm	--	--	No	
105	Cell-ID 20-plex Pd barcode	After thaw	Fluidigm	--	--	No	
106	Cell-ID 20-plex Pd barcode	After thaw	Fluidigm	--	--	No	
108	Cell-ID 20-plex Pd barcode	After thaw	Fluidigm	--	--	No	
110	Cell-ID 20-plex Pd barcode	After thaw	Fluidigm	--	--	No	
113	CD235ab	After Fc block	BioLegend	HIR2	1	Yes	Two antibodies on same channel as dump for platelets/erythrocytes
113	CD61	After Fc block	BioLegend	C1-PL2	1	Yes	Two antibodies on same channel as dump for platelets/erythrocytes
115	CD44	After Fc block	BioLegend	IM77	1	Yes	
139	Unused	--	--	--	--	Yes	Empty channel
140	CD66	After Fc block	BD Biosciences	B1.1	3	Yes	
141	CD16	After Fc block	BioLegend	3G8	1	Yes	
142	CD123	After Fc block	BioLegend	6H6	4	Yes	
143	CD117	After Fc block	Fluidigm	104D2	(1:100)	Yes	
144	CD15	After Fc block	Fluidigm	W6D3	(1:100)	Yes	
145	CD4	After Fc block	Fluidigm	RPA-T4	(1:100)	Yes	
146	CD8a	After Fc block	Fluidigm	RPA-T8	(1:100)	Yes	
147	CD303 (BDCA2)	After Fc block	Fluidigm	201A	(1:100)	Yes	
148	ICOS	After Fc block	BioLegend	C398.4A	2	Yes	
149	BDCA1	After Fc block	BioLegend	L161	2	Yes	
150	FceRIa	After Fc block	Fluidigm	AER-37 (CRA-1	(1:100)	Yes	
151	CD14	After Fc block	Fluidigm	M5E2	(1:100)	Yes	
152	ULBP-2	After Fc block	R&D Systems	165903	0.5	Yes	
153	CD7	After Fc block	Fluidigm	CD7-6B7	(1:100)	Yes	
154	CD163	After Fc block	Fluidigm	GHI/61	(1:100)	Yes	
155	PD-1	After Fc block	Fluidigm	EH12.2H7	(1:100)	Yes	
156	PDGFRb	After Fc block	Fluidigm	18A2	(1:100)	Yes	
157	Epcam	After Fc block	BioLegend	9C4	1	Yes	
158	CD33	After Fc block	Fluidigm	WM53	(1:100)	Yes	
159	CD137 (4-1BB)	After Fc block	BioLegend	4B4-1	2	Yes	
160	CD68	After Perm-S	BioLegend	Y1/82A	1	Yes	
161	CD90	After Fc block	Fluidigm	5E10	(1:100)	Yes	
162	Siglec-8	After Fc block	BioLegend	7C9	2	Yes	
163	CD209	After Fc block	BioLegend	14E367	2	Yes	
164	Arginase-1	After Perm-S	Fluidigm	658922	(1:100)	Yes	
165	MICA/MICB	After Fc block	BioLegend	6D4	4	Yes	
166	pRb	After Perm-S	Fluidigm	J112-906	(1:100)	No	
167	CD38	After Fc block	Fluidigm	HIT2	(1:100)	Yes	
168	CD127 (IL-7Ra)	After Fc block	Fluidigm	A019D5	(1:100)	Yes	
169	CD25	After Fc block	Fluidigm	2A3	(1:100)	Yes	
170	CD3	After Fc block	Fluidigm	UCHT1	(1:100)	Yes	
171	CD304 (BDCA4)	After Fc block	BioLegend	12C2	2	Yes	
172	PD-L2	After Fc block	Fluidigm	24F.10C12	(1:100)	Yes	
173	CD141 (BDCA3)	After Fc block	Fluidigm	1A4	(1:100)	Yes	
174	HLA-DR	After Fc block	Fluidigm	L243	(1:100)	Yes	
175	PD-L1	After Fc block	Fluidigm	29E.2A3	(1:100)	Yes	
176	CD56	After Fc block	Fluidigm	NCAM16.1	(1:100)	Yes	
191	DNA	Final step	Fluidigm	--	--	No	Ir-intercalator
193	DNA	Final step	Fluidigm	--	--	No	Ir-intercalator
194	Viability	Before PFA	Fluidigm	--	--	No	Cisplatin
195	Viability	Before PFA	Fluidigm	--	--	No	Cisplatin
198	Viability	Before PFA	Fluidigm	--	--	No	Cisplatin
209	CD11b	After Fc block	Fluidigm	ICRF44	(1:100)	Yes	

Table S4 – Mouse CyTOF Antibody Panel

Channel	Marker	Staining step	Manufacturer	Clone	Staining Concentration (µg/mL)	Used for clustering	Notes
89	CD45	After Fc block	BioLegend	30-F11	0.5	Yes	
102	Cell-ID 20-plex Pd barcode	After thaw	Fluidigm	--	--	No	
104	Cell-ID 20-plex Pd barcode	After thaw	Fluidigm	--	--	No	
105	Cell-ID 20-plex Pd barcode	After thaw	Fluidigm	--	--	No	
106	Cell-ID 20-plex Pd barcode	After thaw	Fluidigm	--	--	No	
108	Cell-ID 20-plex Pd barcode	After thaw	Fluidigm	--	--	No	
110	Cell-ID 20-plex Pd barcode	After thaw	Fluidigm	--	--	No	
113	B220	After Fc block	BioLegend	RA3-6B2	0.5	Yes	
115	CD11a	After Fc block	UCSF mAb core	M17/4	2	Yes	
139	Ly-6G	After Fc block	UCSF mAb core	1A8	2	Yes	
140	Cleaved PARP	After Perm-S	BD Biosciences	F21-852	8	No	
141	CD38	After Fc block	BioLegend	90	1	Yes	
142	CD49b (a2 integrin)	After Fc block	BioLegend	HMα2	0.13	Yes	
143	CD11c	After Fc block	BD Biosciences	HL3	See notes	Yes	Stained at 2 µg/mL in Figures 6, S6, and S7; 1 µg/mL in all others
144	GFP	After Perm-S	BioLegend	FM264G	2	Yes	
145	CD27	After Fc block	BioLegend	LG.3A10	4	Yes	
146	CD206 (mannose receptor)	After Fc block	BioLegend	C068C2	See notes	Yes	Stained at 2 µg/mL in Figures 6, S6, and S7; 8 µg/mL in all others
147	CD274 (PD-L1)	After Fc block	BioLegend	10F.9G2	1	Yes	
148	CD103 (aE integrin)	After Fc block	BioLegend	2E7	4	Yes	
149	CD152 (CTLA-4)	After Perm-S	BioLegend	UC10-4B9	2	Yes	
150	CD317 (PDCA-1)	After Fc block	BioLegend	927	2	Yes	
151	Ly-6C	After Fc block	BioLegend	HK1.4	0.25	Yes	
152	Ki-67	After Perm-S	eBioscience	SolA15	1	No	
153	CD11b (Mac1)	After Fc block	UCSF mAb core	M1/70	0.5	Yes	
154	Rb (pS807/S811)	After Perm-S	BD Biosciences	J112-906	1	No	
155	CD8a	After Fc block	BioLegend	53-6.7	0.5	Yes	
156	CD4	After Fc block	BioLegend	RM4-5	0.13	Yes	
157	CD3e	After Fc block	BioLegend	17A2	1	Yes	
158	CD279 (PD-1)	After Fc block	BioLegend	29F.1A12	1	Yes	
159	CD31	After Fc block	BioLegend	390	1	Yes	
160	iNOS	After Perm-S	eBioscience	CXNFT	0.5	Yes	160Gd-iNOS was used in all figures other than Figure 6, S6, and S7
160	H-2Ld/H-2Db (MHC-I)	After Fc block	BioLegend	28-14-8	4	Yes	160Gd-MHC-I was used in Figures 6, S6, and S7
161	T-bet	After Perm-S	BD Biosciences	04-46	2	Yes	
162	CD135 (Flt3)	After Fc block	eBioscience	A2F10	8	Yes	
163	CD62L (L-selectin)	After Fc block	BioLegend	MEL-14	8	Yes	
164	CD278 (ICOS)	After Fc block	BioLegend	C398.4A	1	Yes	
165	CD69	After Fc block	BioLegend	H1.2F3	See notes	Yes	Stained at 8 µg/mL in Figures 1D, 1E; 4 µg/mL in all others
166	CD49d (a4 integrin)	After Fc block	BioLegend	R1-2	0.5	Yes	
167	FoxP3	After Perm-S	BioLegend	150D	8	Yes	
168	CD25 (IL-2Ra)	After Fc block	BioLegend	3C7	8	Yes	
169	F4/80	After Fc block	BioLegend	BM8	8	Yes	
170	CD115 (CSF-1R)	After Fc block	BioLegend	AFS98	8	Yes	
171	CD192 (CCR2)	After Fc block	BioLegend	SA203G11	See notes	Yes	Stained FITC-anti-CCR2 (10 µg/mL) followed by 171-anti-FITC (BioLegend clone FIT-22, 2 µg/mL)
172	KLRG1	After Fc block	Tonbo	2F1	0.5	Yes	
173	CD194 (CCR4)	After Fc block	BioLegend	2G12	See notes	Yes	Stained Biotin-anti-CCR4 (10 µg/mL) followed by 173-anti-Biotin (BioLegend clone 1D4-C5, 0.13 µg/mL)
174	CD197 (CCR7)	After Fc block	BioLegend	4B12	8	Yes	
175	CD44	After Fc block	BioLegend	IM7	0.06	Yes	
176	CD90	After Fc block	BioLegend	G7	0.06	Yes	
191	DNA	Final step	Fluidigm	--	--	No	Ir-intercalator
193	DNA	Final step	Fluidigm	--	--	No	Ir-intercalator
194	Viability	Before PFA	Fluidigm	--	--	No	Cisplatin
195	Viability	Before PFA	Fluidigm	--	--	No	Cisplatin
198	Viability	Before PFA	Fluidigm	--	--	No	Cisplatin
209	I-A/I-E (MHC II)	After Fc block	BioLegend	M5/114.15.2	0.06	Yes	

Table S5 – Mouse Tumor Inoculation Details

Cell Line	# of Cells	Injection Volume (uL)	Matrigel?	Route	Figures	Experiment IDs
SB28-Parental	50,000	2	No	Intracerebral	2B, 2D-F	170814
SB28-Parental	500,000	100	No	Subcutaneous	2C-F	180815
SB28-Parental	1,600	2	No	Intracerebral	3B	180123
SB28-Parental	500,000	100	Yes (50% v/v)	Subcutaneous	3C-E	170810, 171129, 181209
SB28-OVA FL	1,000	2	No	Intracerebral	4	180302
SB28-OVA FL	500,000	100	No	Subcutaneous	4	180302
SB28-Parental	1,600	2	No	Intracerebral	5, 6	180301, 180613, 180808, 190424

Table S6 – Mouse Treatment Details

Treatment	Clone	Manufacturer	Catalog Number	Dose (per mouse)	Route	Treatment Schedule	Reference
anti-CD4	GK1.5	BioXcell	BE0003-1	250 µg	i.p.	Days -3, -2, -1, then twice weekly	Smith et al Oncoimmunology 2016 (PMID: 28197381)
anti-CD8a	2.43	BioXcell	BE0061	250 µg	i.p.	Days -3, -2, -1, then twice weekly	Smith et al Oncoimmunology 2016 (PMID: 28197381)
anti-NK1.1	PK136	BioXcell	BE0036	250 µg	i.p.	Days -3, -2, -1, then twice weekly	Smith et al Oncoimmunology 2016 (PMID: 28197381)
anti-CD40L (CD154)	MR-1	BioXcell	BP0017-1	500 µg	i.p.	Twice weekly beginning Day 0	Brasseit et al. Mucosal Immunology 2016 (PMID: 26376366)
anti-CTLA-4	9H10	BioXcell	BE0131	200 µg	i.p.	Every 2-3 days beginning Day 7 unless otherwise specified in the figure	Twyman-Saint Victor et al Nature 2015 (PMID: 25754329)
anti-PD-1	RMP1-14	BioXcell	BE0146	200 µg	i.p.	Every 2-3 days beginning Day 7 unless otherwise specified in the figure	Twyman-Saint Victor et al Nature 2015 (PMID: 25754329)
anti-PD-L1	10F.9G2	BioXcell	BE0101	500 µg then 200 µg	i.p.	In Figure 5 & S5C: 500 ug on Day 4 then 200 ug on Days 7, 10 13; in Figure S5D: 500ug on Day 7 then 200ug on Days 10, 13, 16	Wainwright et al 2014 (PMID 24691018)
Ctrl IgG (mouse IgG2a isotype control for anti-NK1.1)	C1.18.4	BioXcell	BE0085	250 µg	i.p.	Days -3, -2, -1, then twice weekly	Smith et al Oncoimmunology 2016 (PMID: 28197381)
Ctrl IgG (polyclonal Armenian hamster IgG control for anti-CD40L)	--	BioXcell	BP0091	500 µg	i.p.	Twice weekly beginning Day 0	Brasseit et al. Mucosal Immunology 2016 (PMID: 26376366)
Ctrl IgG (hIgG1 control for huFlt3L-Fc)	--	BioXcell	BE0096	10 µg	i.p.	Daily on Day 1 through Day 10	Swee et al 2009 (PMID 19211508), although they used rFLT3L (human) from Amgen
Ctrl IgG (rat IgG1 isotype control for anti-CD25)	--	BioXcell	BE0088	200 µg	i.p.	Days 5 and 7	Vargas et al 2017 (PMID 28410988)
Ctrl IgG (rat IgG2a isotype control for anti-CTLA-4)	--	BioXcell	BE0089	200 µg	i.p.	Every 2-3 days beginning Day 7	
Ctrl IgG (rat IgG2b isotype control for anti-PD-L1, anti-CD4, and anti-CD8)	--	BioXcell	BE0090	500 µg then 200 µg	i.p.	In Figure 5 & S5C: 500 ug on Day 4 then 200 ug on Days 7, 10 13; in Figure S5D: 500ug on Day 7 then 200ug on Days 10, 13, 16	Wainwright et al 2014 (PMID 24691018)
Ctrl IgG (Syrian hamster IgG control for anti-PD-1)	--	BioXcell	BE0087	200 µg	i.p.	Every 2-3 days beginning Day 7	
huFlt3L-Fc	--	BioXcell	BE0098	10 µg	i.p.	Daily on Day 1 through Day 10	Swee et al 2009 (PMID 19211508), although they used rFLT3L (human) from Amgen
Poly-ICLC	--	Oncovir, Inc.	--	20 µg	i.m.	Twice per week from Day 2 through Day 30	Zhu et al 2010 (PMID 20549206)
XRT radiation (1 x 10 Gy)	--	--	--	1 x 10 Gy	--	On Day 10	Zeng et al 2013 (PMID 23462419)
XRT radiation (3 x 8 Gy)	--	--	--	3 x 8 Gy	--	On Days 5, 7, 9	Zeng et al 2013 (PMID 23462419)

Table S7 – Mouse Flow Cytometry Antibody Panel

Figure panel(s)	Fluorophore	Marker	Staining step	Manufacturer	Clone	Staining Concentration (μg/mL)	Catalog Number
4E, S4J-K	BV421	CD45	After Fc block	BioLegend	30-F11	2.5	103134
4E, S4J-K	BV605	CD103	After Fc block	BioLegend	2E7	0.6	121433
4E, S4J-K	PerCP-Cy5.5	I-A/I-E (MHC II)	After Fc block	BioLegend	M5/114.15.2	5	107625
4E, S4J-K	PE	CD8	After Fc block	BioLegend	53-6.7	1.25	100707
4E, S4J-K	PE-Cy7	CD3e	After Fc block	BioLegend	145-2C11	5	100320
4E, S4J-K	APC	H-2Kb bound to SIINFEKL	After Fc block	BioLegend	25-D1.16	10	141605
4E, S4J-K	APC	MHC tetramer (SIINFEKL)	After Fc block	MBL International	--	Diluted 1:12	TB-5001-2
4C-D, S4D-I	BV510	CD4	After Fc block	BD	RM4-5	2	563106
4C-D, S4D-I	BV421	CD3e	After Fc block	BD	145-2c11	2	562600
4C-D, S4D-I	APC-Cy7	CD8	After Fc block	BD	53-6.7	2	557654
4C-D, S4D-I	PE-Cy7	CD45	After Fc block	BioLegend	30-F11	1	103114
4C-D, S4D-I	PE-Dazzle594	Ki67	After Fc block + True-Nuclear permeabilization	BioLegend	16A8	4	652427
4C-D, S4D-I	PE	MHC tetramer (SIINFEKL)	After Fc block	MBL International	--	Diluted 1:50	TB-5001-1
4C-D, S4D-I	APC	MHC tetramer I-Ab OVA 323-339	After Fc block	MBL International	--	Diluted 1:50	TS-M710-2
