# Supplementary figures and images for: Deep immune profiling reveals targetable mechanisms of immune evasion in immune checkpoint inhibitor-refractory glioblastoma
Source: J Immunother Cancer. 2021 Jun 2;9(6):e002181. doi: 10.1136/jitc-2020-002181 (PMC8183210; doi:10.1136/jitc-2020-002181)

Figure S1

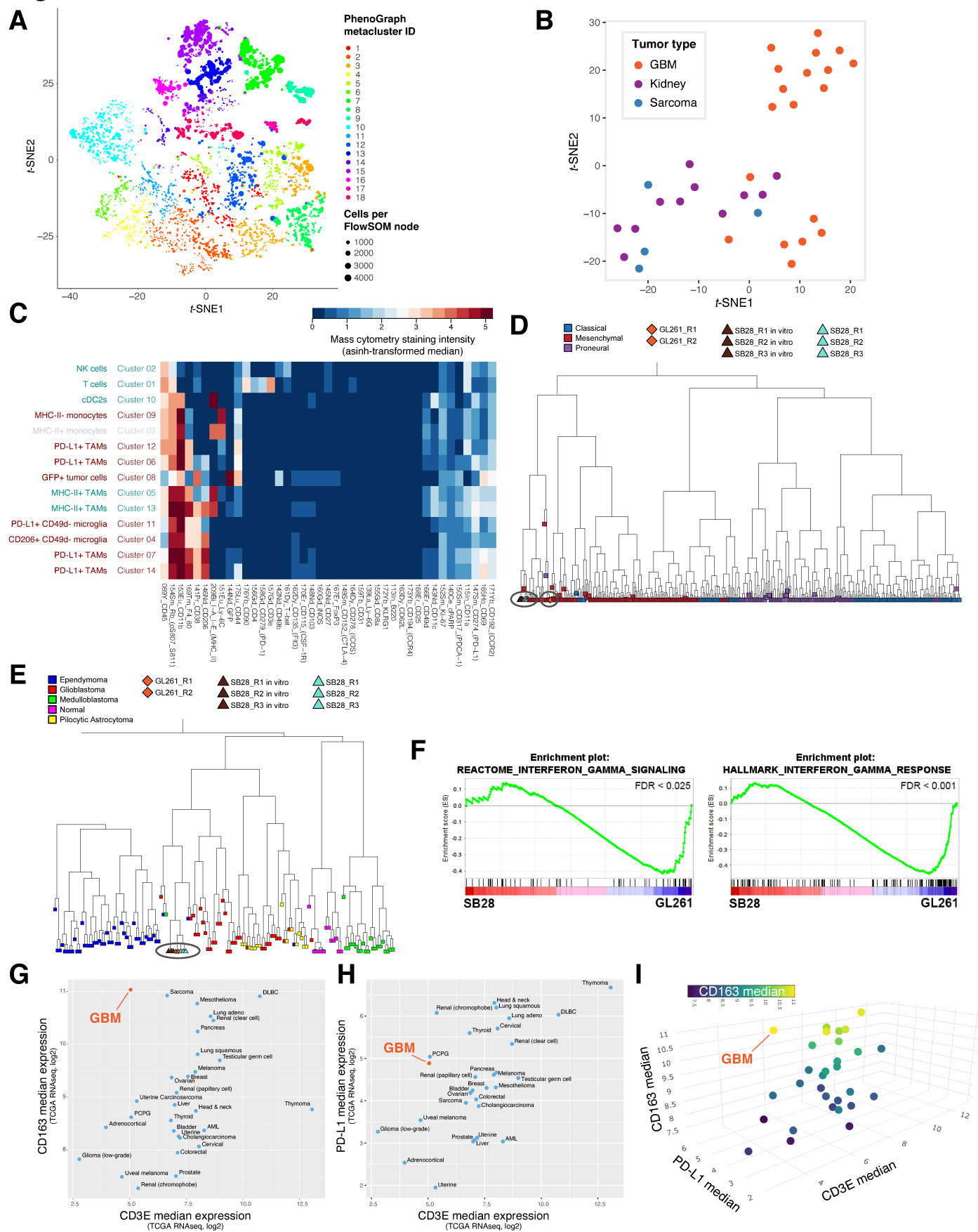

Supplement: Supplementary data [file jitc-2020-002181supp003.pdf]

## Figure S2

A

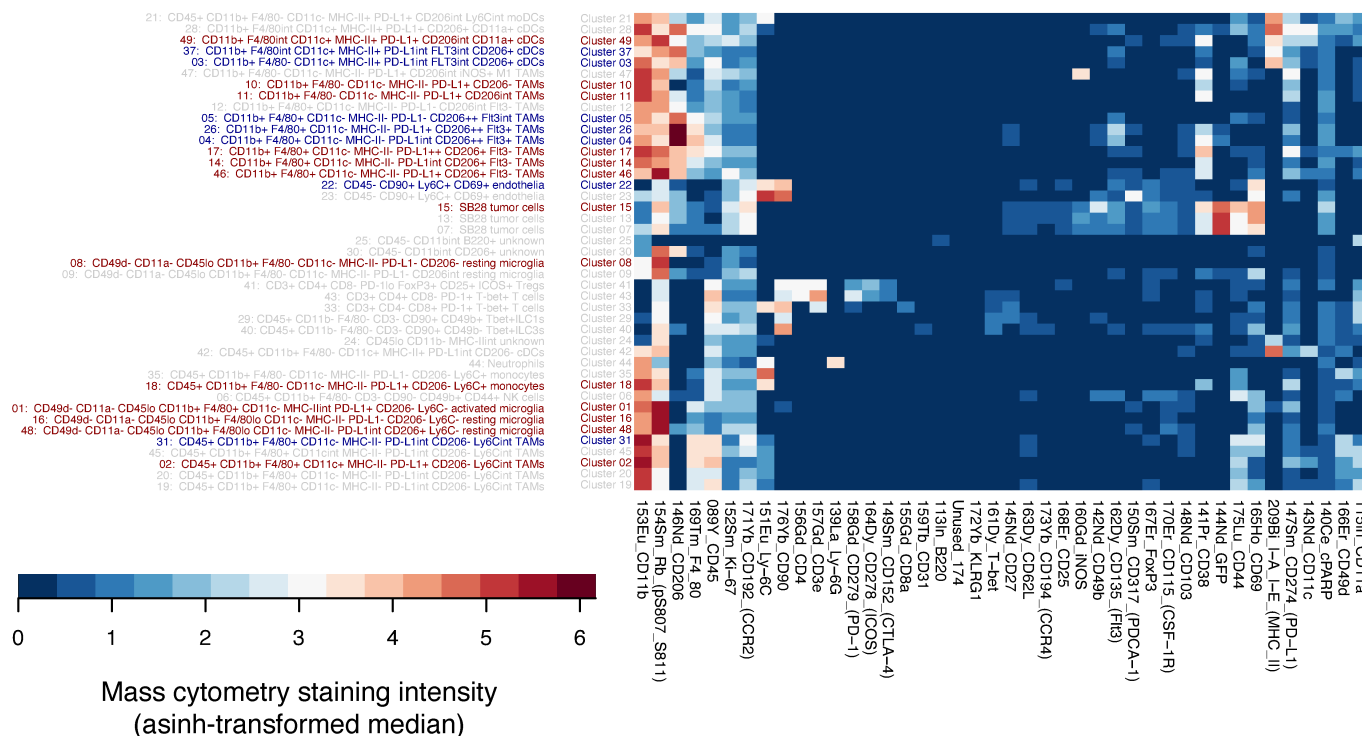

B

Gating strategy for TAM subsets (one representative i.c. tumor is shown):

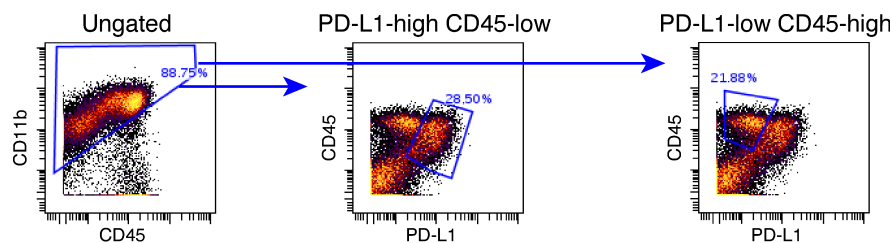

C

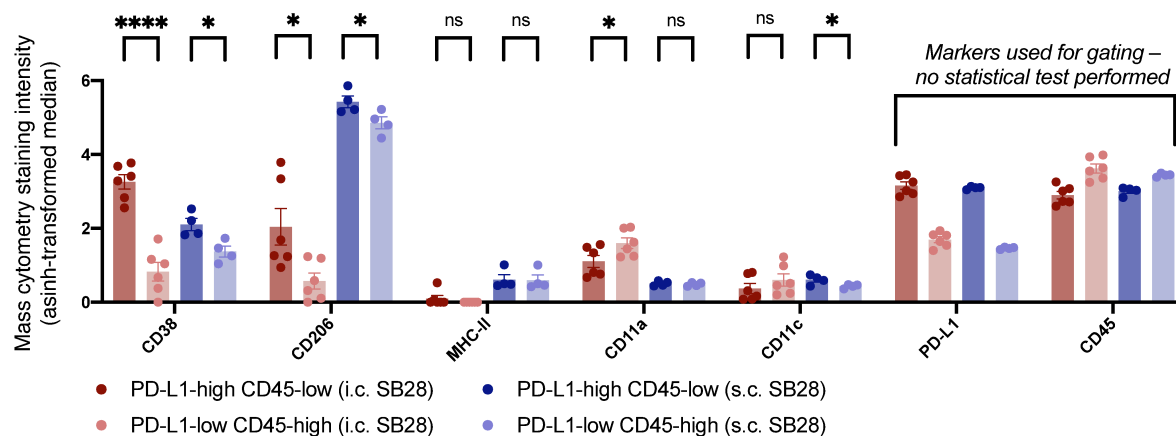

Supplement: Supplementary data [file jitc-2020-002181supp004.pdf]

## Figure S3

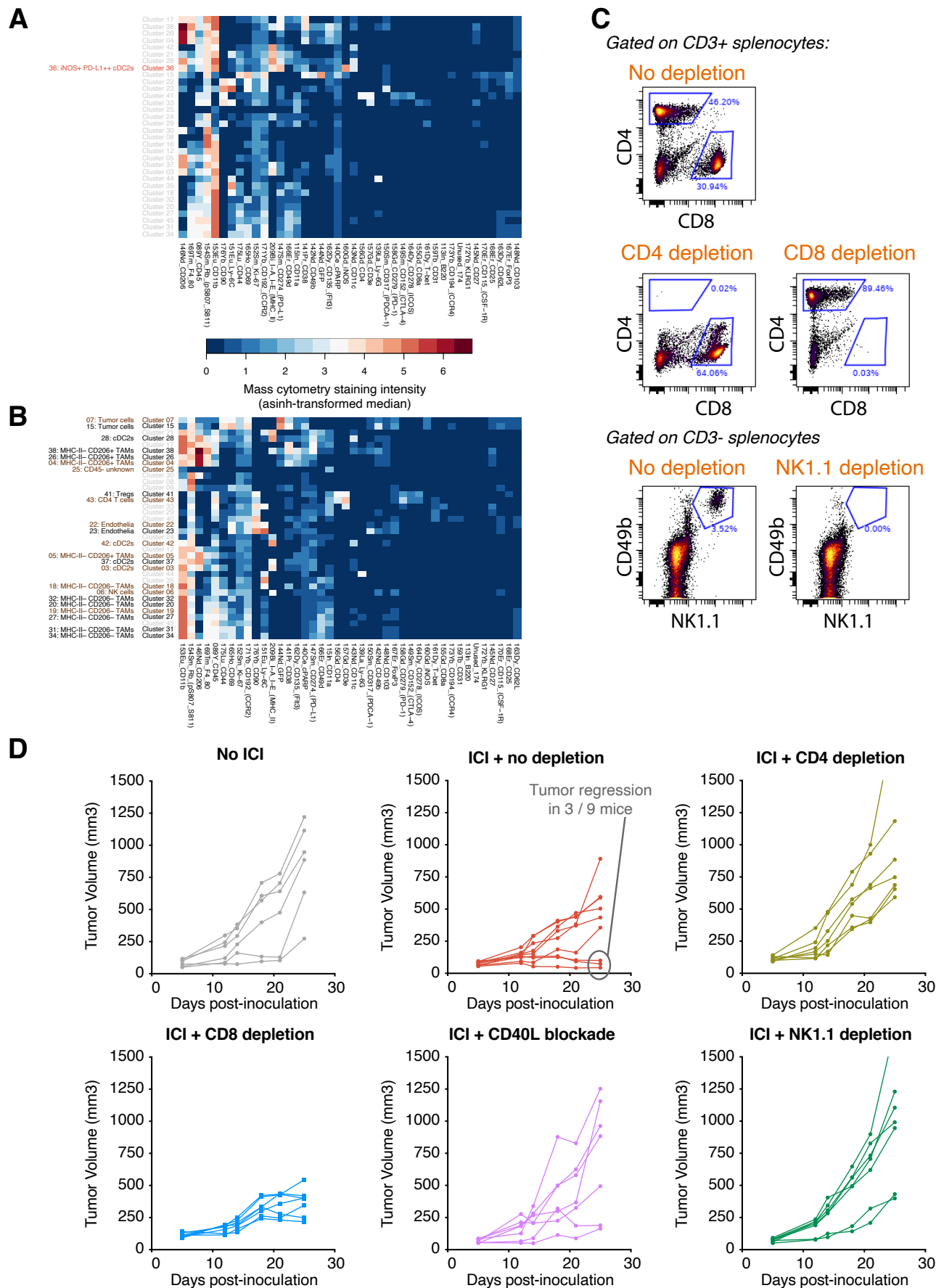

Supplement: Supplementary data [file jitc-2020-002181supp005.pdf]

## Figure S4

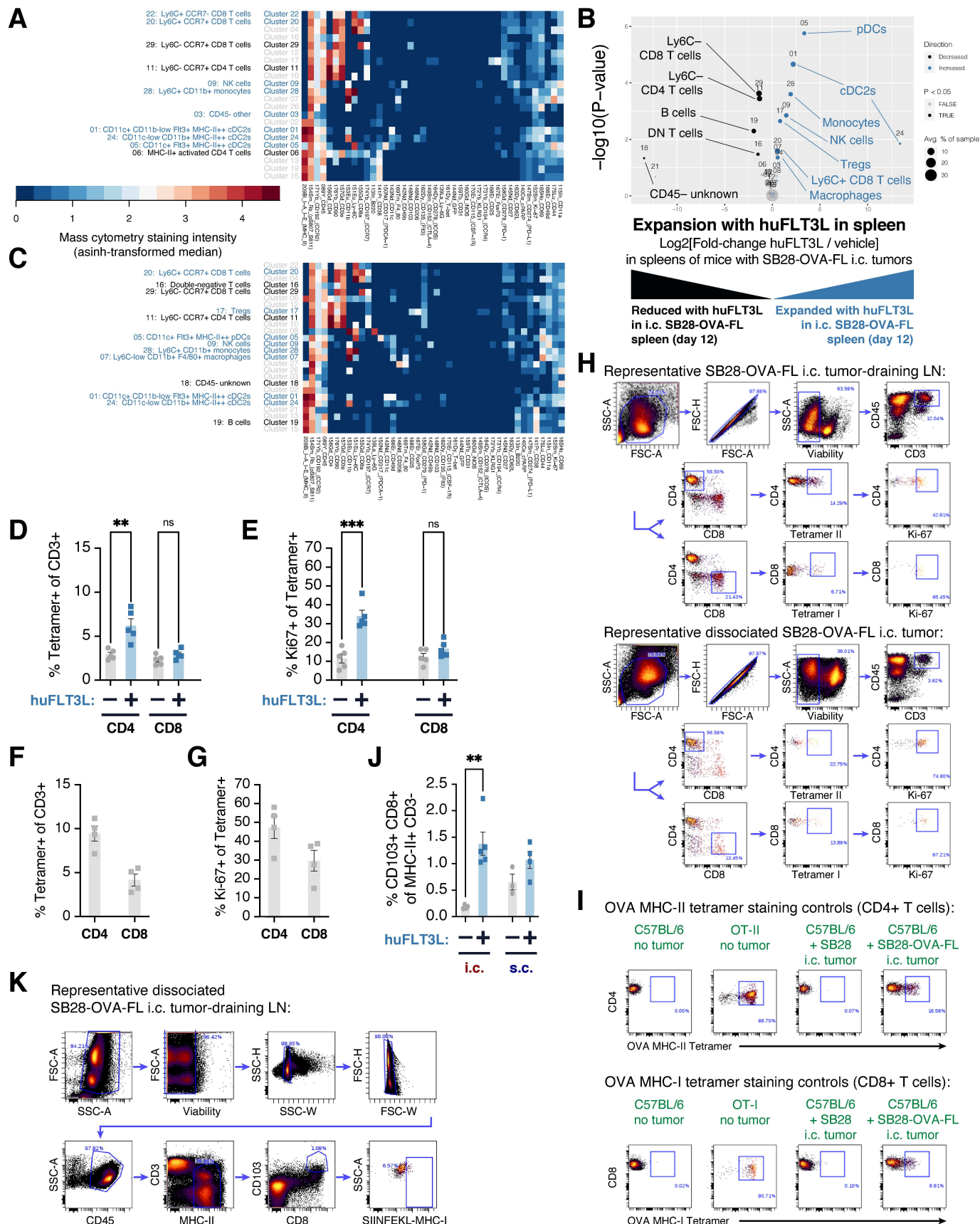

Supplement: Supplementary data [file jitc-2020-002181supp006.pdf]

**Figure S5****A**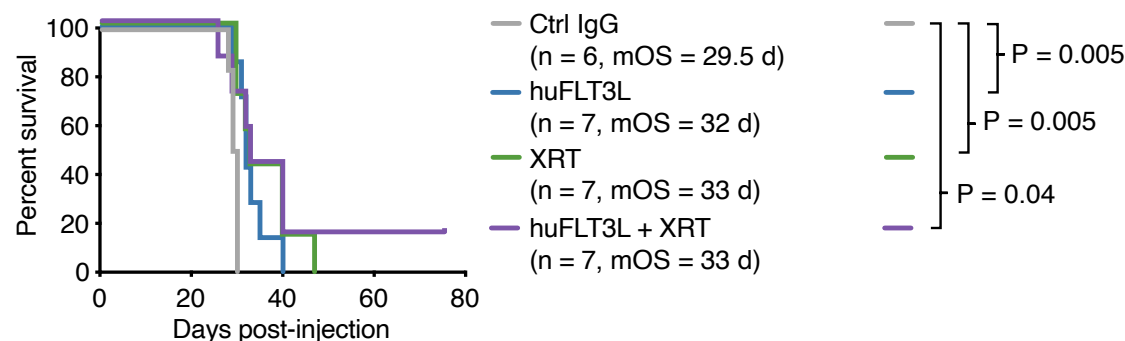**B**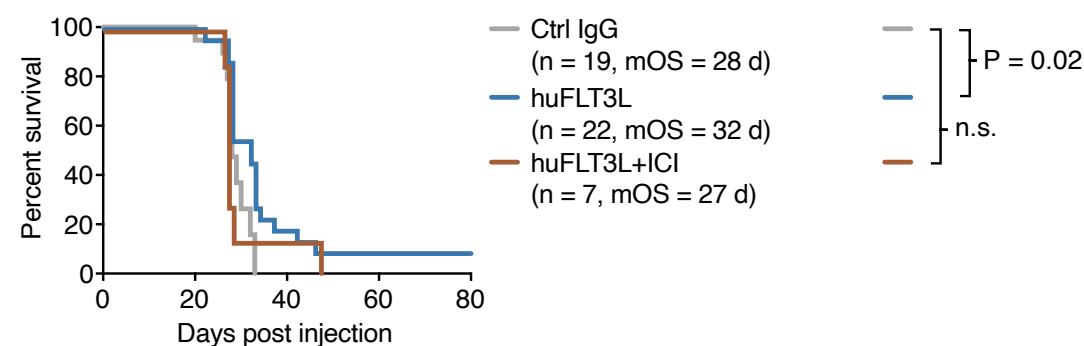**C**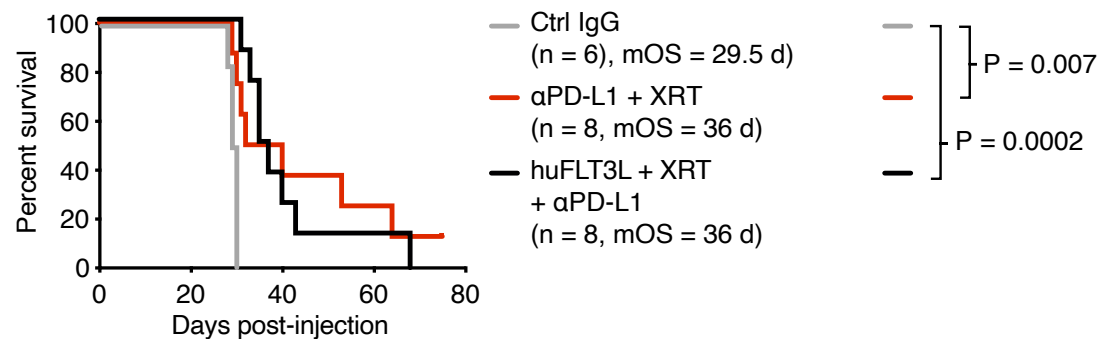**D**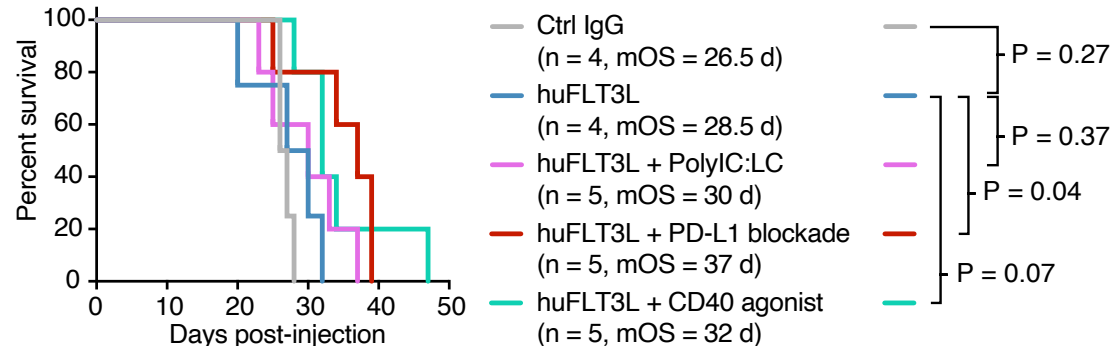

Supplement: Supplementary data [file jitc-2020-002181supp007.pdf]

## Figure S6

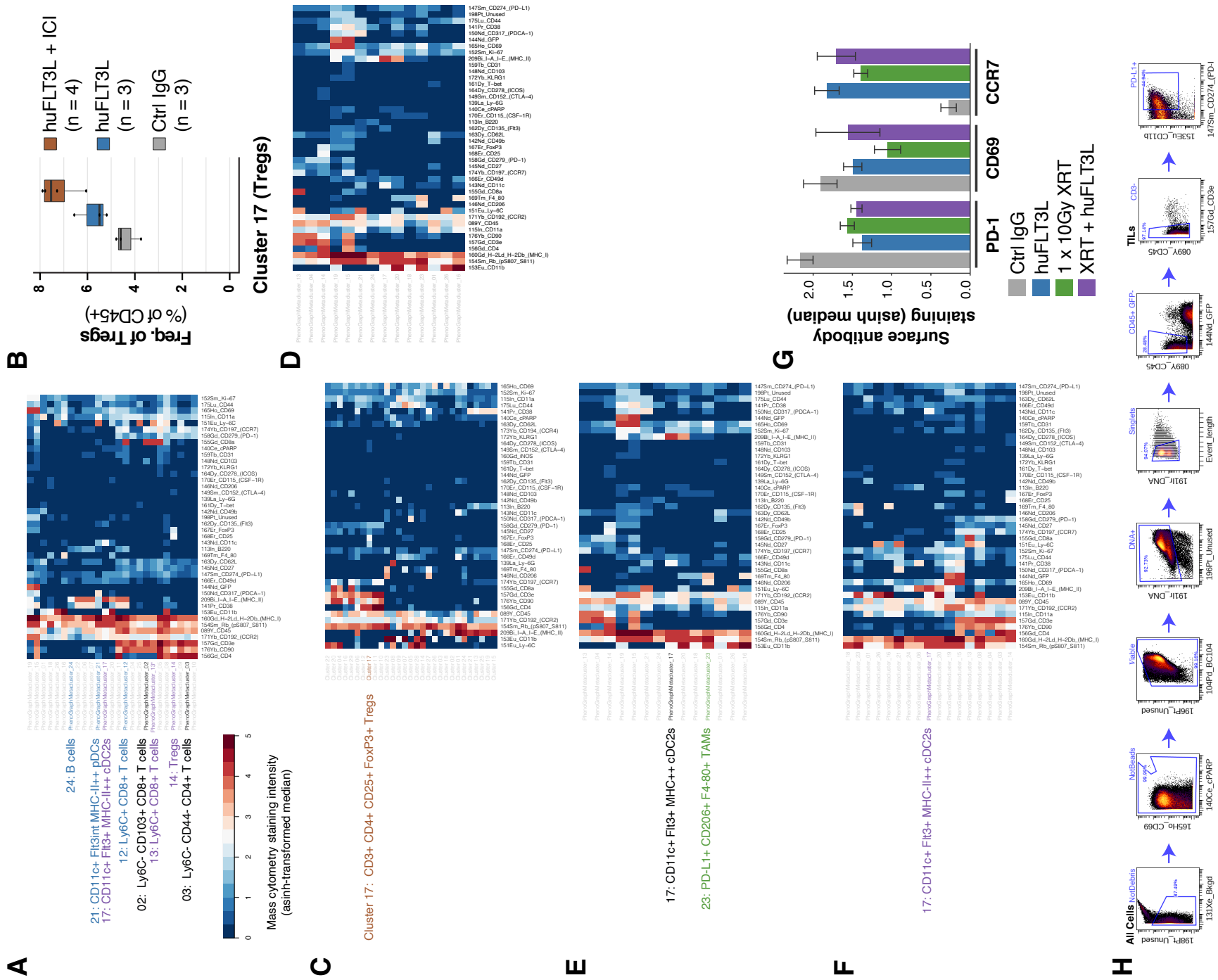

Supplement: Supplementary data [file jitc-2020-002181supp008.pdf]

Cluster 9: PD-L1+ CD206+ F4/80+ TAMs  
P = 3.5e-11

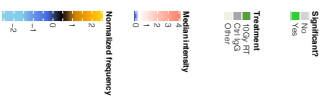

Supplement: Supplementary data [file jitc-2020-002181supp009.pdf]
