# Supplementary material for: Deep immune profiling reveals targetable mechanisms of immune evasion in immune checkpoint inhibitor-refractory glioblastoma
Source: J Immunother Cancer. 2021 Jun 2;9(6):e002181. doi: 10.1136/jitc-2020-002181 (PMC8183210; doi:10.1136/jitc-2020-002181)
Supplement: Supplementary data [file jitc-2020-002181supp010.pdf]

Supplementary Figure Legends

Figure S1.

A) Same as **Figure 1A** except Myeloid CyTOF panel

B) Same as **Figure 1A** except Myeloid CyTOF panel

C) Heatmap of median marker expression in clusters shown in **Figure 1D**

D) Gene expression profiles of GL261 orthotopic tumors (n=2), SB28 orthotopic tumors (n=3), or SB28 cells grown in vitro (n=3) were compared with primary human gliomas from Verhaak et al 2017 by hierarchical clustering.

E) Gene expression profiles of GL261 orthotopic tumors (n=2), SB28 orthotopic tumors (n=3), or SB28 cells grown in vitro (n=3) were compared with primary human brain tumors and normal brain tissue (n = 13) by hierarchical clustering.

F) Gene set enrichment analysis (GSEA) comparing interferon gamma signaling pathway activation in bulk mRNA from GL261 tumors relative to SB28 tumors. FDR: False discovery-rate Q-value.

G) TCGA analysis of human GBM shows an immunosuppressive TME characterized by a higher ratio of M2 TAMs to T cells (CD163:CD3), as compared to other tumor types.

H) TCGA analysis of human GBM shows an immunosuppressive TME characterized by a higher ratio of PD-L1-positive cells to T cells (PD-L1:CD3), as compared to other tumor types.

I) TCGA data of human GBM as in **(F)** and **(G)**, shown as a 3D projection.

Figure S2.

A) Heatmap of median marker expression in clusters shown in **Figure 2D.**

B) Alternative gating strategy for TAM subsets based on CD45 and PD-L1 expression, used in (**C**). Data from the i.c. tumor from **Figure 2E** is shown as a representative sample.

C) Quantification of marker expression by mass cytometry in TAM subsets gated on CD45 and PD-L1. In intracerebral SB28 tumors, the PD-L1-high CD45-low TAM subset expressed significantly more CD38 than the PD-L1-low CD45-high TAM subset.

Figure S3.

A) Heatmap of median marker expression in clusters shown in **Figure 3C**

B) Heatmap of median marker expression in clusters shown in **Figure 3D**. Scale bar as in **(A)**.

C) Flow cytometry of splenocytes from representative mice confirming successful depletion

D) Tumor growth curves of individual mice from ICI depletion cohort

Figure S4.

A) Heatmap of median marker expression in clusters shown in **Figure 4B**

B) Treatment with huFLT3L expands cDC2s, pDCs, and Tregs in the spleens of mice with SB28-OVA-FL tumors at Day 12 post-inoculation. Volcano plot as in **Figure 4B**.

C) Heatmap of median marker expression in clusters shown in **(B)**

D) Treatment with huFLT3L expands OVA-specific T cells in spleen, corresponding to **Figure 4C**

E) Treatment with huFLT3L expands Ki67+ OVA-specific T cells in spleen, corresponding to **Figure 4D**

F) Frequency of OVA tetramer+ CD4 and CD8 T cells (out of CD3+ T cells) in dissociated SB28-OVA-FL tumors at Day 12 post-inoculation.

G) Frequency of proliferating (Ki-67+) T cells (out of OVA tetramer+ T cells) in dissociated SB28-OVA-FL tumors at Day 12 post-inoculation.

H) Gating strategy for OVA MHC tetramer+ and Ki-67+ T cells shown in **Figure 4C-D**

I) T cells were isolated from transgenic OT-I or OT-II mice, or from mice with SB28 parental or OVA-FL tumors. These positive and negative controls confirmed specific staining of OVA MHC tetramer+ T cells using the same reagents shown in **(H).**

J) Treatment with huFLT3L expands cDC1 cells in intracerebral but not subcutaneous SB28-OVA-FL tumors. Frequencies of cDC1 cells (CD103+ CD8+ as a frequency of MHCII+ CD3- events) were quantified by flow cytometry in tumor-draining lymph nodes of intracerebral and subcutaneous SB28-OVA-FL tumors +/- huFLT3L treatment. Tissues were harvested on Day 11 and 12 post-inoculation for s.c. and i.c. tumors, respectively.

K) Gating strategy for cDC1 (CD8+ CD103+ MHC-II+ CD3-) in **(J)** and anti-SIINFEKL-MHC-I in **Figure 4E**.

Figure S5.

A) Kaplan-Meier curves of intracerebral SB28 treated with isotype control IgG (gray), huFLT3L (blue), radiation (XRT, green) or huFLT3L + XRT (purple). Log-rank test P-values and median overall survival are shown.

B) Kaplan-Meier survival curves of intracerebral SB28 treated with isotype control IgG (gray), huFLT3L (blue), or huFLT3L + ICI (brown). Log-rank test P-values and median overall survival are shown.

C) Kaplan-Meier curves of intracerebral SB28 treated with isotype control IgG (gray), double (anti-PD-L1 + XRT, red) or triple-combination immunotherapy (huFLT3L + XRT + anti-PD-L1, black). Log-rank test P-values and median overall survival are shown.

D) Kaplan-Meier survival curves of intracerebral SB28 treated with control IgG (gray), huFLT3L (blue), huFLT3L + PolyIC:LC (pink), huFLT3L + anti-PD-L1 (red), or huFLT3L + CD40 agonist (teal). Log-rank test P-values and median overall survival are shown.

Figure S6.

A) Heatmap of median marker expression in clusters shown in **Figure 6A-B**.

B) In mass cytometry analysis of cervical lymph nodes at endpoint (Day 27-33), Tregs were expanded by huFLT3L alone and even more when combined with anti-PD-1/anti-CTLA-4 (ICI). Box plot shows frequency of Tregs (Cluster 17: CD3+ CD4+ CD25+ FoxP3+) as a percentage of CD45+ cells.

C) Heatmap of median marker expression in Cluster 17 (Tregs), same dataset as **(B)**.

D) Heatmap of median marker expression in clusters shown in **Figure 6C**. No clusters had significantly differential abundance.

E) Heatmap of median marker expression in Cluster 17 (cDC2) and 23 (PD-L1+ TAMs), same dataset as **Figure 6D**.

F) Heatmap of median marker expression in Cluster 17 (cDC2), same dataset as **Figure 6E**.

G) Changes in the surface immunophenotypes of tumor-infiltrating T-cells in SB28 intracerebral tumors treated with FLT3L, XRT or FLT3L + XRT. Values are transformed medians of mass cytometry intensity measurements.

H) Gating strategy for column graph in **(G)**.

Figure S7.

A) The differential abundance analysis from (6D) was repeated using the CATALYST+diffcyt analysis pipeline from Nowicka et al 2019 to compare their performance on an identical dataset (see **Online Supplemental Methods**). The Nowicka pipeline identified a cluster (Cluster 9) that is significantly enriched in RT vs. Ctrl_IgG. The phenotype of this cluster matches the phenotype of Cluster 23 (PD-L1+ CD206+ F4-80+ TAMs) identified with the FlowSOM+PhenoGraph pipeline and shown in **Suppl Figure S6E.**
