# Supplementary material for: Deep immune profiling reveals targetable mechanisms of immune evasion in immune checkpoint inhibitor-refractory glioblastoma
Source: J Immunother Cancer. 2021 Jun 2;9(6):e002181. doi: 10.1136/jitc-2020-002181 (PMC8183210; doi:10.1136/jitc-2020-002181)
Supplement: Supplementary data [file jitc-2020-002181supp011.pdf]

Graphical Abstract

Deep immune profiling reveals targetable mechanisms of immune evasion in immune checkpoint inhibitor-refractory glioblastoma

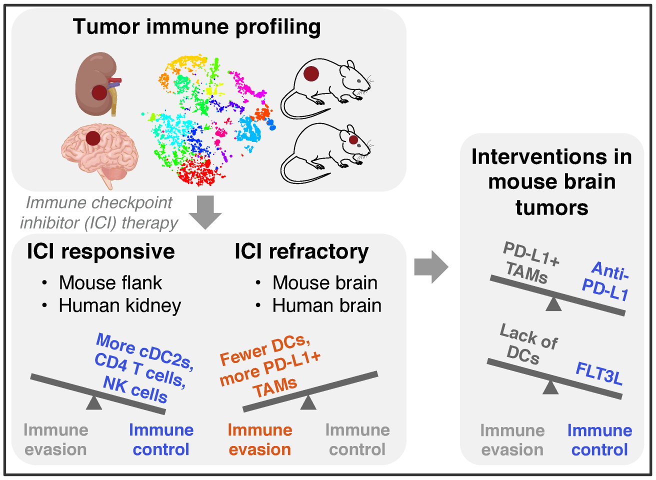

Authors

Erin F. Simonds, Edbert D. Lu, Oscar Badillo, Shokoufeh Karimi, Eric V. Liu, Whitney Tamaki, Chiara Rancan, Kira M. Downey, Jacob Stultz, Meenal Sinha, Lauren K. McHenry, Nicole M. Nasholm, Pavlina Chuntova, Anders Sundström, Vassilis Genoud, Shilpa A. Shahani, Leo D. Wang, Christine E. Brown, Paul R. Walker, Fredrik J. Swartling, Lawrence Fong, Hideho Okada, William A. Weiss, and Mats Hellström

Correspondence

mats.hellstrom@igp.uu.se, hideho.okada@ucsf.edu, waweiss@gmail.com

In Brief

In mice and humans, tumors that were sensitive to immune checkpoint inhibition (ICI) had consistent immunological features. A mouse model of glioma that is refractory to ICI was sensitized by increasing antigen presentation through a variety of approaches.
